# Management of adult-onset Still’s disease with interleukin-1 inhibitors: evidence- and consensus-based statements by a panel of Italian experts

**DOI:** 10.1186/s13075-019-2021-9

**Published:** 2019-12-11

**Authors:** Serena Colafrancesco, Maria Manara, Alessandra Bortoluzzi, Teodora Serban, Gerolamo Bianchi, Luca Cantarini, Francesco Ciccia, Lorenzo Dagna, Marcello Govoni, Carlomaurizio Montecucco, Roberta Priori, Angelo Ravelli, Paolo Sfriso, Luigi Sinigaglia, AOSD Consensus Group

**Affiliations:** 1 Dipartimento di Scienze Cliniche, Internistiche, Anestesiologiche e Cardiovascolari, Rheumatology Unit, Sapienza University of Rome, Rome, Italy; 2 Division of Rheumatology, ASST Gaetano Pini-CTO, Milan, Italy; 3 Rheumatology, Department of Medical Sciences, University of Ferrara and Azienda Ospedaliera-Universitaria di Ferrara, Cona, FE Italy; 4 SC Reumatologia, ASL3 - Azienda Sanitaria Genovese, Genoa, Italy; 5 Department of Medical Sciences, Surgery and Neurosciences, Rheumatology Unit, University of Siena, Policlinico “Le Scotte”, Siena, Italy; 6 Rheumatology, Dipartimento di Medicina di Precisione, Università della Campania “L. Vanvitelli”, Naples, Italy; 7 Unit of Immunology, Rheumatology, Allergy and Rare Diseases (UnIRAR), IRCCS San Raffaele Scientific Institute, Milan, Italy; 8 Department of Rheumatology, IRCCS Policlinico San Matteo Foundation, University of Pavia, Pavia, Italy; 9 Clinica Pediatrica e Reumatologia, Istituto Giannina Gaslini and Università degli Studi di Genova, Genoa, Italy; 10 Rheumatology Unit, Department of Medicine, University of Padua, Padua, Italy

**Keywords:** Adult-onset Still’s disease, Anakinra, Canakinumab, Interleukin-1, Rilonacept, Still’s disease, Systemic juvenile idiopathic arthritis

## Abstract

**Background:**

Adult-onset Still’s disease (AOSD) is a rare inflammatory condition characterized by fever, rash, and arthritis. Because of its rarity, clinical trials are inherently small and often uncontrolled. Our objective was to develop recommendations for the use of interleukin (IL)-1 inhibitors in the management of patients with AOSD, based on the best evidence and expert opinion.

**Methods:**

A panel of 10 experts (9 rheumatologists and 1 pediatrician) was established. The first step was dedicated to a comprehensive literature review and development of statements. Two separate literature searches were performed on the MEDLINE (Pubmed), EMBASE, and BIOSIS databases through April 2018 to identify (1) differences and similarities between AOSD and pediatric Still’s disease (systemic juvenile idiopathic arthritis [SJIA]) and (2) the efficacy and safety of IL-1 inhibitors in AOSD treatment. In the second step, the statements were submitted in a Delphi process to a panel of 67 rheumatologists. Consensus threshold was set at 66%: positive, > 66% of voters selected scores 3 to 5; negative, > 66% of voters selected scores 1 or 2. In the third step, the voting results were analyzed, and the statements were finalized.

**Results:**

Eleven statements were developed. Forty-six of 67 rheumatologists (72%) participated in the Delphi process. A positive consensus was reached after the first round of voting and was full (> 95%) on the majority of statements. A large consensus was achieved in considering AOSD and SJIA as the same disease. The use of anti-IL-1 therapies in refractory patients was considered quite safe and effective both as the first and as a subsequent line of biologic treatment, especially in systemic patients. Because of the lack of head-to-head comparisons, a different profile of efficacy among IL-1 inhibitors could not be established. There was a large consensus that failure of the first IL-1 inhibitor does not preclude response to another one. The lack of studies comparing early versus late treatment did not allow to draw conclusions; however, data from SJIA suggest a better response in early treatment.

**Conclusions:**

The Delphi method was used to develop recommendations that we hope will help clinicians in the management of patients with AOSD refractory to conventional therapies.

## Introduction

Still’s disease is a rare systemic inflammatory disorder that can develop both in adults and children and is characterized by a wide spectrum of manifestations, including a typical triad of symptoms: daily spiking fever, polyarthritis, and a characteristic salmon-colored skin rash [1]. Still’s disease was first defined in children, whereas the adult form was described in 1971, following the identification of a clinical condition in adults with clinical and laboratory features similar to that observed in children [1, 2]. The childhood form of the disease is currently termed systemic juvenile idiopathic arthritis (SJIA), while the adult form is known as adult-onset Still’s disease (AOSD) [1]. Despite attempts to identify serological biomarkers, there is currently no universally standardized biomarker for AOSD [3]. The diagnosis of AOSD is still challenging and frequently based on the exclusion of other conditions, with consequent diagnostic delays [4, 5]. Although many experts view SJIA and AOSD as the same disease, the true relationship between the two conditions has long been a matter of debate [1]. Based on the presence of predominant “systemic” or “chronic articular” manifestations, two different subtypes of AOSD have been proposed [6]. These two subtypes are characterized by distinct serological profiles, with evidence of a major pro-inflammatory state in the systemic form compared to the articular form [6–10].

The pathophysiology of Still’s disease is largely unknown, but recent advances have identified interleukin (IL)-1β as a crucial inflammatory mediator [6, 11]. Both SJIA and AOSD have been shown to respond dramatically to the IL-1β blockade [12]. Currently available IL-1 inhibitors include anakinra, canakinumab, and rilonacept. Anakinra, a human recombinant IL-1 receptor antagonist, is approved in the European Union (EU) for Still’s disease (SJIA and AOSD) with active systemic features of moderate to high disease activity, or after the failure of conventional treatments [non-steroidal anti-inflammatory drugs (NSAIDs) or glucocorticoids] [13]. Canakinumab, a human monoclonal anti-human IL-1β antibody, is approved in the EU for the treatment of active AOSD and SJIA, following an inadequate response to NSAIDs and systemic corticosteroids [14]; according to the US FDA label, canakinumab is indicated for active SJIA [15]. Rilonacept, an IL-1 trap fusion protein, is currently not licensed for use in Still’s disease either in Europe or the USA [16, 17].

To date, there are no guidelines for the management of Still’s disease, and the treatment of this condition is mostly empirical and challenging for many clinicians. Because of the rarity of the disease, very few clinical studies are available. Specifically, there is only one randomized controlled trial (RCT) on the use of anakinra in patients with AOSD [18], and data supporting the efficacy/safety of canakinumab are only available from two RCTs performed in patients with SJIA [19]. Based on the similarities with SJIA, the European Medicines Agency (EMA) has now approved the use of canakinumab in patients with AOSD [20]. To address the lack of evidence-based guidance on the management of AOSD, a panel of ten Italian physicians with expertise in the management of Still’s disease convened a meeting in January 2018 with the goal of addressing unresolved issues and reaching consensus about the management of patients with AOSD using IL-1 inhibitors. The issues addressed by the panel were mainly related to the relationship between SJIA and AOSD, the efficacy and safety of IL-1β blockade in AOSD, the optimal timing for treatment initiation, and the efficacy of these agents in the articular versus the systemic form of AOSD. After a comprehensive review of the literature, the panel used a Delphi process to develop consensus statements on the management of AOSD. This article presents the outcomes of this process as a summary of the consensus statements and supportive literature.

## Methods

### Design

A panel composed of 9 Italian rheumatologists and 1 pediatrician expert in pediatric rheumatology (scientific board of the project) were chosen based on their clinical experience and expertise in the treatment of Still’s disease (Table 1). Expertise to qualify for the scientific board was defined as having treated more than 15 patients with AOSD in the previous 15 years and being an author on at least 1 published article on AOSD. In addition, the panel members were from diverse areas of Italy in order to gain geographic representation from across the country.

**Table 1:** Membership of the scientific board and Delphi respondents

Participating experts	Number	Specialty, *n* (%)	Hospital-based, *n* (%)^1^	University-based, *n* (%)^1^	Geographical origin, *n* (%)
Scientific board	10	Rheumatology, 9 (90) Pediatrics, 1 (10)	6 (60)	8 (80)	Northern Italy, 7 (70) Central Italy, 2 (20) Southern Italy, 1 (10)
Bibliographic board	4	Rheumatology (100)	2 (50)	3 (75)	Northern Italy, 3 (75) Central Italy, 1 (25)
Delphi respondents	49	Rheumatology (100)	22 (45)	43 (88)	Northern Italy, 20 (41) Central Italy, 18 (37) Southern Italy, 11 (22)

^1^Percentages do not add to 100 because some experts are both hospital- and university-based

The scientific panel met in Milan on January 23, 2018, to discuss the issues related to the treatment of AOSD with anti-IL-1 inhibitors and to plan the development of a series of expert opinion- and evidence-based statements addressing these issues. During the first meeting, a comprehensive search of the literature was planned to gather the available evidence on the relationship between SJIA and AOSD and on IL-1β blockade in AOSD. Because a limited number of publications were expected, especially with regard to the efficacy/safety of anti-IL-1 agents in AOSD, the scientific board decided to base the statements on a combination of available evidence and consensus procedures, which had to be conducted through a Delphi process. The Delphi technique is widely used in medical research to generate consensus on controversial or insufficiently documented issues [21–25]. The degree of reliability of a given statement depends on the percentage of consensus achieved through voting by a panel of experts. For issues on which no consensus was reached, the voting process was repeated after a plenary discussion among the experts or the review of supporting literature.

### Consensus development process

The consensus development process was undertaken between April 2018 and October 2018 and consisted of three steps: (i) comprehensive literature review and writing of statements; (ii) evaluation of statements by a panel of rheumatologists, who were asked to express their agreement or disagreement on each statement; and (iii) assessment of voting results and finalization of the statements.

The first step involved two meetings. The first meeting was held in Milan on April 3, 2018, and was attended by the scientific board and by 4 additional rheumatologists (AB, SC, MM, TS), who were responsible for the search and selection of the literature (bibliographic board). The aim of this meeting was to design the strategy of the literature search. During the second meeting, held on September 10, 2018, the bibliographic board presented the results of the literature search. Based on the results of the literature review, 11 statements were drafted. The level of evidence (range 1–5) and the grade of recommendation (range A–D) were assigned to each statement according to the Oxford Centre for Evidence-Based Medicine [26].

In the second step of the consensus process (the Delphi portion), the statements were circulated electronically to a panel of 67 rheumatologists from 31 Italian rheumatology centers; since AOSD is a rare disease requiring specialist management, these rheumatologists were all members of the Working Group of Systemic Autoinflammatory Diseases of the Società Italiana Reumatologia (SIR; Italian Society of Rheumatology) and were from all areas of Italy (Table 1). The rheumatologists were invited to express their agreement or disagreement about each statement, using a 5-point Likert scale (1 = maximal disagreement, 2 = disagreement, 3 = agreement, 4 = strong agreement, 5 = absolute [total] agreement). The consensus threshold was set at 66%. A positive consensus was considered to be reached when > 66% of voters selected the scores 3 to 5 and negative consensus to be reached when > 66% of voters selected the scores 1 or 2. If consensus was not reached, the statements were submitted to the second round of discussion and review.

At the third and final step of the consensus process, the scientific board and the bibliographic board analyzed the voting results and finalized the statements during a final meeting held in Milan on October 16, 2018.

### Literature search strategy

Two separate literature searches were undertaken. These literature searches were performed on three databases (MEDLINE via Pubmed, Embase, and BIOSIS Previews). No start date for the literature search was defined, but the end date was April 20, 2018.

First, a systematic literature review was performed to identify reports describing the similarities and differences of SJIA and AOSD. A search strategy was developed using Mesh (or Emtree) terms and free-text words identifying AOSD and SJIA (Additional file 1: Table S1). The reviewers selected the literature pertaining to the similarities and differences of AOSD and SJIA, by limiting the analysis to studies or case series addressing clinical features, outcomes, laboratory parameters, response to treatment, genetic profile, and classification criteria.

A second systematic literature review was performed to identify articles describing the efficacy and safety of IL-1β blockade in AOSD. Four specific clinical questions were considered relevant in relation to the treatment of AOSD with IL-1 inhibitors: (1) efficacy and safety of IL-1 inhibitors in general, (2) comparative efficacy and safety of different IL-1 inhibitors, (3) comparison of early versus late treatment with IL-1 inhibitors, and (4) relative efficacy of IL-1 inhibitors in patients with a systemic versus chronic articular pattern of AOSD. Each clinical question was phrased according to the Patient, Intervention, Control, Outcomes (PICO) strategy for study selection. The main database searches were complemented by a manual search of proceedings from the European League Against Rheumatism (EULAR) and American College of Rheumatology (ACR) congresses of the previous 2 years (2016 and 2017) and also by scrutinizing the reference lists of included articles. A search strategy was developed using Mesh (or Emtree) terms and free-text words identifying AOSD, along with potential terms for anti-IL-1 agents (Additional file 1: Table S2). The potential terms for the anti-IL-1 agents included brand names and generic names for the individual IL-1 inhibitors, as well as generic terms for interleukin-1 and “anti,” “inhibit,” “block,” or “antagonist.” The study selection and data extraction were performed by two independent reviewers. For the evaluation of efficacy outcomes, only controlled clinical trials, cohort studies, or case series were assessed. For safety outcomes, case reports were also included.

## Results

### Literature search results

#### Relationship between AOSD and SJIA

The literature search identified 332 publications (Additional file 1: Table S1). After the selection of studies corresponding to inclusion criteria, 30 publications comparing AOSD and SJIA were identified. These covered similarities and differences in clinical features, outcomes, laboratory parameters, and response to treatment [4, 27–47], genetic profile [48–50], and classification criteria for AOSD and SJIA [51–56]. Two relevant reviews on AOSD were also identified [1, 6].

#### Efficacy and safety of IL-1 inhibition in AOSD

The search of the literature about IL-1 inhibition in AOSD (Additional file 1: Table S2) identified 358 publications; among them, 27 publications other than case reports were selected as relevant to the clinical questions being investigated (Table 2).

**Table 2:** Publications reporting on the treatment of AOSD with IL-1 inhibitors

Study	Design	Patients	IL-1 treatment
Fitzgerald et al. [57]	Case series	4 patients with refractory AOSD	Anakinra
Kalliolias et al. [58]	Case series	4 patients with refractory AOSD	Anakinra
Kötter et al. [59]	Case series	4 patients with refractory AOSD	Anakinra
Lequerre et al. [60]	Multicenter, retrospective observational study	35 patients (20 SJIA, 15 AOSD)	Anakinra
Franchini et al. [8]	Retrospective chart review	49 patients with AOSD; 6 treated with biologics	Anakinra (*n* = 5)
Henderson et al. (abstract) [61]	Prospective, open-label, dose escalation study	5 patients with AOSD	Rilonacept
Naumann et al. [62]	Case series	8 patients with refractory AOSD (3 with systemic disease, 5 with articular disease)	Anakinra
Laskari et al. [63]	Case series	25 patients with refractory AOSD	Anakinra
Riera et al. [64]	Retrospective review	41 patients; 7 treated with biologics	Anakinra (*n* = 5)
Nordstrom et al. [18]	Open, multicenter RCT	22 patients with AOSD	Anakinra (*n* = 12) or DMARD (*n* = 10)
Quartuccio et al. [65]	Retrospective, observational study	36 patients with AOSD	Anakinra (*n* = 10)
Giampietro et al. [66]	Retrospective, observational study	28 patients with AOSD	Anakinra
Iliou et al. [67]	Retrospective observational study	44 patients with AOSD; 10 treated with anakinra	Anakinra
Gerfaud-Valentin et al. [68]	Retrospective observational study	57 patients with AOSD; 6 treated with anakinra	Anakinra
Hong et al. [69]	Meta-analysis	8 studies, 134 patients with AOSD	Anakinra
Maria et al. [6]	Case series	8 patients with refractory AOSD treated with biologics; 4 treated with IL-1 inhibitors	Anakinra, canakinumab
Cavalli et al. [70]	Retrospective observational study	20 patients with AOSD; 16 treated with anakinra, as first biologic option; eventually all patients received anakinra	Anakinra
Ortiz-Sanjuán et al. [71]	Multicenter, open-label, retrospective study	41 patients with AOSD refractory to standard immunosuppressive therapy and to other biologics	Anakinra
Palmou et al. (abstract) [72]	Multicenter, open-label study	75 patients with AOSD refractory to standard immunosuppressive therapy and to other biologics	Tocilizumab (*n* = 34) or anakinra (*n* = 41)
Rossi-Semerano et al. [73]	Retrospective, observational study	189 patients (35 AOSD, 27 SJIA)	Anakinra, canakinumab
Lenert and Yao [35]	Case series	7 patients with AOSD and MAS	Anakinra (*n* = 5)
Sfriso et al. [74]	Multicenter, retrospective observational study	245 patients with AOSD	58 patients (23.7%) treated with biologics; anakinra as the first-line biologic (*n* = 31) or as the second-line biologic (*n* = 4)
Toz et al. (abstract) [75]	Retrospective analysis of database	24 patients with refractory AOSD; 7 treated with anakinra	Anakinra
Colafrancesco et al. [76]	Multicenter retrospective observational study	140 patients with refractory AOSD; all treated with anakinra; 4 switched to canakinumab after anakinra failure	Anakinra, canakinumab
Junge et al. [77]	Comprehensive literature review with data summaries	45 articles, > 150 patients with AOSD	Anakinra, canakinumab, rilonacept
Feist et al. [33]	Subgroup analysis of pooled data from 4 SJIA studies	29 older adolescents/younger adults with SJIA, considered representative of patients with AOSD	Canakinumab
Neel et al. [78]	Multicenter retrospective observational study and systematic literature review	20 patients with AOSD admitted to ICU; 5 patients received anakinra	Anakinra

*AOSD* adult-onset Still’s disease, *DMARD* disease-modifying anti-rheumatic drug, *ICU* intensive care unit, *IL-1* interleukin-1, *MAS* macrophage activation syndrome, *RCT* randomized controlled trial, *SJIA* systemic juvenile idiopathic arthritis

These articles included 1 RCT [18, 33, 61], 1 post hoc analysis of pooled study results [31], 1 prospective open-label trial [54], 5 nationwide surveys [60, 66, 71, 73, 74], 17 retrospective observational studies or case series [6, 8, 57–59, 62–64, 67, 68, 70, 72, 76, 78], 1 meta-analysis [69], and 1 comprehensive review [77]. A total of 60 case reports, mostly related to the use of anakinra were also identified; 7 case reports described the use of canakinumab in AOSD [79–85], and 3 case reports documented the use of rilonacept in AOSD [83, 86, 87]; the complete list of case reports can be viewed in Additional file 1: Table S3 [27, 59, 79–134].

### Delphi process and consensus development

Based on the review of the literature and on personal clinical experience, the scientific board developed 11 statements concerning the relationship between AOSD and SJIA and the role of IL-1 inhibition in the treatment of AOSD. The statements (English translation) are shown in Table 3 along with the results of the Delphi voting.

**Table 3:** Statements and results of Delphi survey

Statement	Agreement (%)	Level of evidence (range)	Grade of recommendation
1.1 Adult Onset Still’s Disease (AOSD) and Systemic Juvenile Idiopathic Arthritis (SJIA) show substantial similarities in terms of clinical manifestations, laboratory features, response to treatment and, possibly, genetic background.	94	3b–4	C
1.2 Adult onset Still’s Disease (AOSD) and Systemic Juvenile Idiopathic Arthritis (SJIA) may be considered the same disease. Differences in the rate of clinical manifestations could be related to the age of onset.	88	5	D
2.1 There is evidence that IL-1 inhibition represents an effective therapeutic approach in AOSD refractory to conventional treatment and/or other biologics.	96	2a	B
2.2 In AOSD, treatment with IL-1 inhibitors is effective on different clinical and laboratory parameters and displays a significant steroid sparing effect in most patients. Therapeutic response is rapid and sustained over time.	100	2a	B
2.3 IL-1 inhibitors are effective in the treatment of AOSD-related Macrophage Activation Syndrome (MAS), although cases of MAS occurring during treatment with these drugs are reported.	98	2b–4	C
2.4 IL-1 inhibitors have an overall satisfactory safety profile in AOSD. Among adverse events, infections have been reported. Treatment with anakinra has been associated with frequent injection site reactions and occasionally severe cases of hepatotoxicity, reversible after treatment withdrawal.	98	2b–5	C
3.1 A different profile of efficacy among IL-1 inhibitors cannot be established, because head-to-head comparisons are lacking and available data deal with the use of canakinumab and rilonacept almost exclusively after anakinra failure.	100	5	D
3.2 Considering available data on efficacy, failure of the first IL-1 inhibitor does not preclude the achievement of a therapeutic response with another IL-1 inhibitor.	88	5	D
4.1 There are no available studies comparing the efficacy of the treatment with IL-1 inhibitors in early versus late stages of AOSD. Data from SJIA suggest that early treatment with IL-1 inhibitors is associated with a better therapeutic response.	96	5	D
4.2 Indirect data show that IL-1 inhibitors can be effective in AOSD both in first and subsequent lines of biologic treatment.	98	2b	B
5.1 Some data suggest that IL-1 inhibitors may be more effective on systemic rather that chronic articular manifestations of AOSD.	98	2b	C

*AOSD* adult-onset Still’s disease, *DMARD* disease-modifying anti-rheumatic drug, *IL-1* interleukin-1, *SJIA* systemic juvenile idiopathic arthritis

A total of 49 rheumatologists out of the 67 invited by the scientific board participated in the Delphi process (72% participation rate). The threshold for positive consensus (> 66% agreement) was reached on each statement during the first round of voting, and no additional Delphi rounds were required. Consensus exceeded 95% for the majority of statements.

### Relationship between AOSD and SJIA

Statement 1.1. Adult Onset Still’s Disease (AOSD) and Systemic Juvenile Idiopathic Arthritis (SJIA) show substantial similarities in terms of clinical manifestations [3b], laboratory features [3b], response to treatment [3b] and, possibly, genetic background [4]. [Grade C]

Statement 1.2. Adult Onset Still’s Disease (AOSD) and Systemic Juvenile Idiopathic Arthritis (SJIA) may be considered the same disease. Differences in the rate of clinical manifestations could be related to the age of onset [5]. [Grade D]

As shown in Table 4, despite a few differences, there is a substantial overlap between AOSD and SJIA in clinical characteristics, laboratory parameters, outcomes, and response to treatment. Among the eight studies comparing clinical features (Table 4), the presence of a higher incidence of sore throat in adults seems to be the only relevant clinical difference [32, 36, 38, 44]. A single small report suggests a higher rate of amyloidosis in AOSD, potentially in line with the older age of AOSD patients [32]. One study reported a higher frequency of polyarthritis in AOSD [4], yet most studies indicate similar articular involvement [32, 33, 36, 38, 41, 44] with evidence of progressive and erosive arthritis in both adults and children [4, 32, 36]. Although children are more likely to develop an intermittent articular pattern, arthritis seems to occur with similar frequency in SJIA and AOSD [4, 38]. However, worse outcomes have been described in patients with pediatric onset, presumably related to the longer disease duration [36]. Concerning the overall course of the disease, there also seems to be no difference between SJIA and AOSD [4, 32]. In most studies, response to treatment is also similar [4, 32, 33, 37, 38, 44]; better articular outcomes in patients with AOSD than in those with SJIA have been reported in only one study [36]. Taken together, the clinical evidence supports the statement that AOSD and SJIA may be regarded as the same disease, and the few differences in the frequency of signs and symptoms could be related to the age of the affected individual at the time of their first encounter with potential disease-triggering factors [1].

**Table 4:** Reports describing common features shared by AOSD and SJIA or differences between the two conditions

Study	Patients	Similarities	Differences
Clinical features			
Bywaters [2]	AOSD, *n* = 14 SJIA, NA	Adult patients with the same clinical features of SJIA: “Still disease” of the adults?	–
Cabane et al. [32] (retrospective chart review)	AOSD, *n* = 8 SJIA, *n* = 10	No significant differences in initial systemic manifestations or in joint lesions	Amyloidosis restricted to AOSD (*n* = 3)
Tanaka et al. [44] (in Japanese; abstract in English)	AOSD, *n* = 19 SJIA, *n* = 26	Fever and arthritis in all patients Polyarthritis, 77% SJIA versus 53% AOSD	Sore throat more frequent in AOSD (AOSD 68% versus SJIA 19%)
Ohta and Yamaguchi [41] (in Japanese; abstract in English)	AOSD, *n* = 92 SJIA, *n* = 11	91–93% clinical items statistically not different between SJIA and AOSD	–
Uppal et al. [4] (retrospective analysis)	AOSD, *n* = 31 SJIA, *n* = 23	Similar incidence of fever, sore throat, lymphadenopathy, and splenomegaly	Rash and hepatomegaly more common in SJIA Polyarthritis more common in AOSD
Luthi et al. [37] (retrospective review)	AOSD, *n* = 10 SJIA, *n* = 9	Similar presentation at onset (systemic and articular manifestations)	–
Lin et al. [36] (retrospective analysis)	AOSD, *n* = 21 SJIA, *n* = 24	Similar presentation at onset (systemic and articular manifestations)	Sore throat more frequent in AOSD
Pay et al. [38] (multicenter retrospective review)	AOSD, *n* = 95 SJIA, *n* = 25	No differences in the pattern of fever and localization of skin rash	Higher frequency of fever, rash, myalgia, weight loss, and sore throat in AOSD
Response to treatment/clinical outcome			
Cabane et al. [32]	AOSD, *n* = 8 SJIA, *n* = 10	The same treatments Poor articular prognosis in both conditions	–
Tanaka et al. [44]	AOSD, *n* = 19 SJIA, *n* = 26	The same articular prognosis with the same treatments	–
Uppal et al. [4]	AOSD, *n* = 31 SJIA, *n* = 23	Similar clinical outcomes	–
Luthi et al. [37]	AOSD, *n* = 10 SJIA, *n* = 9	Similar clinical outcomes	–
Lin et al. [36]	AOSD, *n* = 21 SJIA, *n* = 24	–	Better articular outcome in AOSD than SJIA
Pay et al. [38]	AOSD, *n* = 95 SJIA, *n* = 25	The same treatments in both conditions	–
Feist et al. [33] (subgroup analysis of pooled data from 4 SJIA studies with canakinumab)	AOSD, *n* = 29 SJIA, *n* = 272	The same response to treatment with canakinumab	–
Laboratory parameters			
Tanaka et al. [44]	AOSD, *n* = 19 SJIA, *n* = 26	Similar initial laboratory data	Serum iron levels more elevated in AOSD than SJIA
Uppal et al. [4]	AOSD, *n* = 31 SJIA, *n* = 23	Similar laboratory profiles	–
Luthi et al. [37]	AOSD, *n* = 10 SJIA, *n* = 9	Similar laboratory profiles Ferritin levels elevated both in SJIA and AOSD	–
Lin et al. [36]	AOSD, *n* = 21 SJIA, *n* = 24	Similar laboratory profiles	Ferritin levels more elevated in AOSD than in SJIA
Pay et al. [38]	AOSD, *n* = 95 SJIA, *n* = 25	Similar laboratory profiles	Liver dysfunction and neutrophilia more common in AOSD than in SJIA
Hashimoto et al. [39] (in Japanese; abstract in English)	AOSD, *n* = 7 SJIA, *n* = 4	IL-2 receptor elevated in sera of AOSD and SJIA patients compared to controls	–
Bae et al. [29]	AOSD, *n* = 37 SJIA, NA	S100A12 (calcium-binding protein) elevation in sera of AOSD compared to controls (as described in SJIA)	–
Kudela et al. [47] (abstract)	AOSD, *n* = 30 SJIA, *n* = 20	Highly elevated IL-18 serum levels in both active AOSD and SJIA	–
Genetic profile			
Miller et al. [48]	AOSD, *n* = 16 SJIA, *n* = 35	–	HLA-DR4 more involved in SJIA HLA-DR7 more involved in AOSD
Cush et al. [50] (ACR-ARHP abstract)	AOSD, *n* = 21 SJIA, NA	Genomic profiling in AOSD patients similar to studies in SJIA patients	–
Nirmala et al. [49]	AOSD, *n* = 17 SJIA, NA	Similar genes upregulated/downregulated by treatment with IL-1 inhibitors in AOSD compared to studies in SJIA patients	–
Classification criteria			
Talesnik et al. [43] (retrospective analysis in Spanish; abstract in English)	AOSD, *n* = 7 SJIA, *n* = 14	The same proportion of patients in each form of disease course (monocyclic systemic form, polycyclic systemic form, monocyclic chronic joint form, polycyclic chronic joint form)	–
El Hamshary et al. [54] (retrospective cross-sectional study)	AOSD, NA SJIA, *n* = 30	23/30 SJIA patients fulfilled Yamaguchi criteria and 20/30 ILAR criteria	–
Kumar et al. [52] (retrospective chart review)	AOSD, NA SJIA, *n* = 31	23/31 SJIA patients fulfilled Yamaguchi criteria and 18/31 ILAR criteria	–
Oliveira Ramos et al. [55] (analysis of Portuguese rheumatic disease registry)	AOSD, NA SJIA, *n* = 66	35/66 SJIA patients fulfilled criteria for AOSD	–
Yang et al. [53] (retrospective review)	AOSD, *n* = 169 SJIA, NA	ILAR criteria for SJIA can identify AOSD patients at risk of relapse, MAS, and ICU	–
Debach et al. [56] (abstract)	AOSD, NA SJIA, *n* = 17	42% of patients with SJIA fulfilled Yamaguchi criteria for AOSD	–

*AOSD* adult-onset Still’s disease, *ICU* intensive care unit, *IL* interleukin, *ILAR* International Leagues of Associations of Rheumatology, *MAS* macrophage activation syndrome, *SJIA* systemic juvenile idiopathic arthritis

The similarity of clinical features is not the sole evidence supporting the notion that AOSD and SJIA may be the same disease. The genetic profiles show similarities in the upregulation of genes involved in the IL-1 signaling pathway (e.g., IL-1β, IL-1 receptor accessory protein, IL-1RN, and IL-1 receptors 1 and 2) and downregulation of genes regulating proliferation and immune function (e.g., *AKTCD24*, *CD28CD3D*, *CD6CD69*, *CDC25B*, and *CDC7*) [49]. There may be differences in the HLA genotype between SJIA and AOSD. In particular, compared to the healthy population, an association with the HLA-DR4 gene has been found only in SJIA [48], whereas the HLA-B14 and HLA-DR7 genes were shown to be associated with AOSD [48].

To date, the diagnosis of SJIA is based on the International League of Associations for Rheumatology (ILAR) criteria [135, 136], whereas AOSD is diagnosed according to the Yamaguchi criteria [137]. However, there is increasing evidence that either set of criteria can be applied to both groups of patients. This evidence lends further support to the possibility that AOSD and SJIA represent a continuum of the same condition. Yang and colleagues [53] found good concordance between ILAR and Yamaguchi criteria in patients with AOSD; moreover, fulfilling ILAR criteria in patients with AOSD seems to be predictive for a worse outcome. On the contrary, SJIA patients meet Yamaguchi criteria more commonly than ILAR criteria [52, 54]. A possible explanation is that although arthritis is a key criterion for the diagnosis of SJIA (ILAR criteria), this feature is often absent, particularly at disease onset [52]. On the other hand, according to the Yamaguchi criteria, arthritis is not a mandatory criterion for AOSD [137]. Notably, a revision of the ILAR classification criteria for SJIA has been recently proposed, which makes them more similar to the Yamaguchi criteria [138]. The overall goal of this revision is to identify more homogeneous clinical subgroups of JIA patients and to distinguish the forms of chronic arthritis seen only in children from those that also occur in adult patients. In the newly proposed classification criteria, SJIA is considered equivalent to AOSD, and an adaptation of the Yamaguchi criteria has been chosen to classify SJIA. According to the proposal, arthritis is no longer required to classify SJIA, thereby recognizing that as in AOSD, patients with SJIA may have systemic features without arthritis. These preliminary criteria will be formally validated through a multinational prospective data collection [138].

### IL-1 inhibition in AOSD

Statement 2.1. There is evidence that IL-1 inhibition represents an effective therapeutic approach in AOSD refractory to conventional treatment and/or other biologics [2a]. [Grade B]

Statement 2.2. In AOSD, treatment with IL-1 Inhibitors is effective on different clinical and laboratory parameters and displays a significant steroid-sparing effect in most patients [2a]. Therapeutic response is rapid and sustained over time. [Grade B]

Statement 2.3. IL-1 inhibitors are effective in the treatment of AOSD-related Macrophage Activation Syndrome (MAS) [4], although cases of MAS occurring during treatment with these drugs are reported [2b]. [Grade C]

Statement 2.4. IL-1 Inhibitors have an overall satisfactory safety profile in AOSD [2b]. Among adverse events, infections have been reported [2b]. Treatment with anakinra has been associated with frequent injection-site reactions [2b] and occasionally severe cases of hepatotoxicity, reversible after treatment withdrawal [5]. [Grade C]

Based on our review of the literature, therapies targeting IL-1 (anakinra, canakinumab, and rilonacept) are significantly effective in patients with AOSD refractory to conventional treatment (Table 5) [6, 18, 33, 35, 60–68, 70–73, 75, 76, 78]. Among the 20 studies reported in Table 5, the effectiveness of anti-IL-1 therapies in AOSD ranged from 50 to 100% (median 83.3%); the rate of remission ranged from 22.2 to 100% (median 70%) and the treatment failure rate from 0 to 50% (median 16.7%). Data on remission must be interpreted with caution since this disease state was not defined in some studies [6, 62, 64, 68, 75] and was defined variably in others [18, 33, 60, 63, 66, 70, 73, 76]. One of the lowest rates of remission (22.2%) was reported in a study in which remission was defined purely on the basis of articular manifestations (i.e., 100% improvement in American College of Rheumatology response criteria) [33], while the highest rates (80–100%) were reported in studies that did not provide a definition of remission [6, 62, 64, 68]. In studies requiring complete recovery of systemic and articular symptoms, as well as normalization of inflammatory biomarkers, the rate of remission varied between 14.2 and 70% [18, 66, 70, 73, 76]. The most stringent definition of remission was provided by Cavalli and colleagues [70], who required the absence of systemic and articular manifestations, the normalization of acute phase reactants (C-reactive protein and erythrocyte sedimentation rate), and a reduction in corticosteroid dosage (≥ 50% for at least 2 months). Using this definition, 70% of patients receiving anakinra achieved remission [70].

**Table 5:** Efficacy of anti-IL agents (anakinra, canakinumab, and rilonacept) in refractory patients with AOSD: data from retrospective observational studies, nationwide survey, and clinical trials

Reference	Design	Study drug	Mean follow-up (months)	Number	Effective (%)	Remission (%)	Failure (%)
Naumann et al. [62]	RO	Anakinra	26.8	8	100	87.5	0
Laskari et al. [63]	RO	Anakinra	12	25	96	84	4
Riera et al. [64]	RO	Anakinra	112.85	5	100	100	0
Quartuccio et al. [65]	RO	Anakinra	22	10	90	NA	10
Iliou et al. [67]	RO	Anakinra	NA	10	100	NA	0
Gerfaud-Valentine et al. [68]	RO	Anakinra	27.8	6	83.3	83.3	16.6
Maria et al. [6]	RO	Anakinra	24	5	100	80	0
Maria et al. [6]	RO	Canakinumab	30	1	100	100	0
Cavalli et al. [70]	RO	Anakinra	60	20	80	70	20
Palmou et al. (abstract) [72]	RO	Anakinra	15.5	41	73	NA	26.8
Lenert and Yao [35]	RO	Anakinra	NA	5	100	NA	0
Toz et al. (abstract) [75]	RO	Anakinra	NA	7	NA	61	NA
Colafrancesco et al. [76]	RO	Anakinra	12	140	81.4	14.2	18.5
Colafrancesco et al. [76]	RO	Canakinumab	12	4	75	NA	25
Neel et al. [78]	RO	Anakinra	NA	5	80	NA	20
Lequerre et al. [60]	Nationwide survey	Anakinra	14.3	15	86.6	73.3	13.3
Giampietro et al. [66]	Nationwide survey	Anakinra	23	28	57	42.8	21.4
Rossi-Semerano et al. [73]	Nationwide survey	Anakinra	NA	35	60	55.8	40
Rossi-Semerano et al. [73]	Nationwide survey	Canakinumab	NA	2	50	50	50
Ortiz-Sanjuan et al. [71]	Nationwide survey	Anakinra	12	41	82.9	NA	17
Nordstrom et al. [18]	Clinical trial (RCT)	Anakinra	6	12	91.6	50	8.3
Henderson et al. (abstract) [61]	Clinical trial (POL dose escalation)	Rilonacept	24	5	60	NA	40
Feist et al. [33]	Clinical trial (post hoc analysis)	Canakinumab	3	29	83.3	22.2	16.7
Median global evaluation					83.3	70	16.65

*abs* conference abstract, *AOSD* adult-onset Still’s disease, *NA* not available, *POL* prospective open-label, *RCT* randomized controlled trial, *RO* retrospective observational

Our data are in agreement with a previous review of the literature performed by Junge and colleagues in 2017 [77]; this review reported that anti-IL-1 agents were associated with high rates of full remission (55–75%) and of full or partial remission (91–100%) [77]. The consistent response to all three anti-IL-1 agents, regardless of the different mechanisms by which they block IL-1β, is a further demonstration of the involvement of IL-1β in the pathogenesis of AOSD.

According to our review of the literature, in most patients with AOSD, it is possible to significantly reduce the dosage of concomitant corticosteroids during treatment with anti-IL-1 therapy [18, 60, 62, 63, 66, 67, 70, 71, 76]. Moreover, corticosteroid withdrawal was achieved in 17 to 48% of patients [18, 60, 63, 70, 76]. This result is consistent with a pooled analysis of published data on IL-1 inhibitors in AOSD, which reported an overall rate of corticosteroid discontinuation of 36.9% (95% CI 24.0–52.0%) [69].

Data on the efficacy of anti-IL1 agents in the treatment of arthritis in patients with AOSD are still controversial. During treatment with anakinra, a significant reduction in the proportion of AOSD patients with arthritis has been reported [60, 63, 71, 76]. A reduction in DAS28 score after 85 days of therapy with canakinumab has also been demonstrated [33]. However, despite such improvements, in some cases, a complete resolution of arthritis could not be achieved [71, 76]. In contrast, systemic manifestations of AOSD, such as fever and rash, showed rapid and sustained disappearance during IL-1 inhibitor therapy [6, 18, 33, 60, 62–68, 70, 71, 73, 76, 78].

Based on our analysis, the most frequently reported adverse events during IL-1 inhibitor treatment are injection site reactions (ISRs), which develop in 10 to 66.6% of patients receiving anakinra (Table 6) [18, 62, 63, 66, 70, 71, 76, 97]. The largest retrospective observational study reported a 20% incidence of ISRs in patients treated with anakinra [76].

**Table 6:** Safety of anti-IL agents (anakinra, canakinumab, and rilonacept) in refractory patients with AOSD: data from retrospective observational studies, nationwide survey, and clinical trials

Study	Design	Study drug	Adverse event, no. of patients
Injection site reactions (ISR)			
Naumann et al. [62]	RO	Anakinra	2/8 (25%)
Laskari et al. [63]	RO	Anakinra	5/25 (20%)
Gerfaud-Valentine et al. [68]	RO	Anakinra	“Some”/6
Cavalli et al. [70]	RO	Anakinra	2/20 (10%)
Colafrancesco et al. [76]	RO	Anakinra	28/140 (20%)
Giampietro et al. [66]	Nationwide survey	Anakinra	“Some”/28
Ortiz-Sanjuan et al. [71]	Nationwide survey	Anakinra	6/41 (14.6%)
Nordstrom et al. [18]	Clinical trial (RCT)	Anakinra	8/12 (66.6%)
Diffuse cutaneous reactions (rash/urticaria/eczema)			
Laskari et al. [63]	RO	Anakinra	3/25 (12%)
Quartuccio et al. [65]	RO	Anakinra	3/10 (30%)
Toz et al. (abstract) [75]	RO	Anakinra	1/7 (14.2%)
Colafrancesco et al. [76]	RO	Anakinra	12/140 (8.5%)
Lequerre et al. [60]	Nationwide survey	Anakinra	2/15 (13.3%)
Ortiz-Sanjuan et al. [71]	Nationwide survey	Anakinra	2/41 (4.8%)
Feist et al. [33]	Clinical trial (post hoc analysis)	Canakinumab	13/31 (41.9%)
Infections (mild and severe)			
Laskari et al. [63]	RO	Anakinra	6/25 mild (24%); 1/25 severe (4%)
Cavalli et al. [70]	RO	Anakinra	2/20 mild (10%)
Colafrancesco et al. [76]	RO	Anakinra	4/140 mild (2.8%); 2/140 severe (1.4%)
Lequerre et al. [60]	Nationwide survey	Anakinra	4/15 mild (26.6%); 1/15 severe (6.6%)
Ortiz-Sanjuan et al. [71]	Nationwide survey	Anakinra	3/41 mild (7.3%); 2/41 severe (4.8%)
Rossi-Semerano et al. [73]	Nationwide survey	Anakinra	2/35 severe (5.7%)
Henderson et al. [61]	Clinical trial (POL dose escalation)	Rilonacept	2/5 severe (40%)
Feist et al. [33]	Clinical trial (post hoc analysis)	Canakinumab	21/31 mild (67.7%); 2/31 severe (6.4%)
Other adverse events			
Quartuccio et al. [65]	RO	Anakinra	2/10 (20%) (thrombocytopenia)
Colafrancesco et al. [76]	RO	Anakinra	2/140 (1.4%) (thrombocytopenia) 1/140 (0.7%) (leukopenia) 1/140 (0.7%) (lymphoproliferative disorders)
Lequerre et al. [60]	Nationwide survey	Anakinra	1/15 (6.6%) (Hip osteonecrosis)
Ortiz-Sanjuan et al. [71]	Nationwide survey	Anakinra	3/41 (7.3%) (leukopenia) 1/41 (2.4%) (myopathy)
Feist et al. [33]	Clinical trial (post hoc analysis)	Canakinumab	18/31 (58%) (GI disorders) 10/31 (32.2%) (respiratory)
Macrophage activation syndrome			
Colafrancesco et al. [76]	RO	Anakinra	6/140 (4.2%)
Colafrancesco et al. [76]	RO	Canakinumab	1/4 (25%)
Rossi-Semerano et al. [73]	Nationwide survey	Anakinra	1/35 (2.8%)
Henderson et al. [61]	Clinical trial (POL dose escalation)	Rilonacept	1/5 (20%)
Feist et al. [33]	Clinical trial (post hoc analysis)	Canakinumab	3/31 (9.6%)

*AOSD* adult-onset Still’s disease, *POL* prospective open-label, *RCT* randomized controlled trial, *RO* retrospective observational

In addition to ISRs, diffuse cutaneous reactions, such as skin rash, urticaria and, in some cases, eczema, have been reported during treatment with anakinra or canakinumab (Table 6) [33, 60, 63, 65, 71, 75, 76]. Other common adverse events include infections and gastrointestinal disorders [33, 60, 61, 63, 70, 71, 73, 76]. The rate of severe infections is quite low, with a reported frequency between 1.4 and 6.6% in patients treated with anakinra and 6.4% in patients treated with canakinumab [33, 60, 61, 63, 70, 71, 73, 76]. In patients receiving anakinra or canakinumab, mild infections, most commonly respiratory tract infections, are generally more frequent than severe infections [33, 60, 61, 63, 70, 71, 73, 76]. In terms of the risk of infection, treatment with anti-IL-1 agents seems to have an acceptable safety profile.

Other less frequent adverse events are reported in Table 6 [33, 60, 65, 71, 76]. Although rare, severe adverse events, mainly represented by major infections, may occur. Specifically, mycobacterial infections, viral infections (cytomegalovirus, varicella zoster), and pneumonia have been reported [33, 61, 71, 73, 76]. Anakinra has also been associated with occasional cases of severe hepatotoxicity that is reversible after treatment withdrawal [89, 112, 139].

Instances of MAS have been reported during treatment with anti-IL-1 agents (anakinra, canakinumab, and rilonacept) (Table 6) [33, 61, 73, 76, 79]. However, because MAS is a frequent complication of AOSD, it is impossible to attribute these events with certainty to the treatment.

Despite limited data, anakinra appears relatively safe during pregnancy [117, 140]. Smith and Chambers [140] reported pregnancy outcomes in five women (three with AOSD and two adults with pediatric-onset SJIA) who received anakinra during the third trimester of pregnancy. All five women delivered live infants at term; two pregnancies had complications (oligohydramnios in both, accompanied by pregnancy-induced hypertension in one) [140]. One infant had a low birth weight (2419 g), but none had major long-term complications or malformations [140]. Similarly, Fischer-Betz and colleagues reported successful pregnancy outcomes in two women treated with anakinra (one who started treatment during pregnancy and the other who received anakinra throughout pregnancy) [117, 140]. Neither woman chose to breastfeed during anakinra therapy.

Evidence on pregnancies in patients exposed to IL-1 inhibitors has substantially increased with the publication of a large case series (43 pregnancies) by Youngstein and colleagues [141]. This article provided the first reports of pregnancy outcome in women treated with canakinumab, as well as reporting on outcomes in women treated with anakinra. The data show that the 2 agents are safe, and most infants were born healthy. One woman with an active disease during 2 pregnancies experienced 2 miscarriages, the first during treatment with anakinra and the second under canakinumab. One case of unilateral renal agenesis was also reported in an infant exposed to anakinra during pregnancy. In this study, data regarding the outcome of paternal IL-1 inhibitor intake were also available. Specifically, all the infants born from fathers treated with IL-1 inhibitors at conception (6 with anakinra and 5 with canakinumab) were healthy and did not have congenital or developmental abnormalities [141].

Taken together, the available data indicate that IL-1 inhibitor therapy is a valid treatment in patients with AOSD who fail to improve with conventional therapies. Response to IL-1 inhibitors is rapid and sustained, and this form of treatment allows patients to reduce their dependence on corticosteroids. The overall safety profile of IL-1 inhibitors is also generally favorable.

### Efficacy and safety of the various IL-1 inhibitors in AOSD

Statement 3.1. A different profile of efficacy among IL-1 inhibitors cannot be established, because head-to-head comparisons are lacking and available data regarding the use of canakinumab and rilonacept are almost exclusively after anakinra failure [5]. [Grade D]

Statement 3.2. Considering available data on efficacy, failure of the first IL-1 inhibitor does not preclude the achievement of a therapeutic response with another IL-1 inhibitor [5]. [Grade D]

There are no head-to-head studies comparing the available anti-IL1 agents in patients with AOSD. Because of its original indication in rheumatoid arthritis, anakinra has been on the market for longer than the other compounds and therefore has been the most extensively investigated in patients with AOSD. Canakinumab and rilonacept are generally used in patients who have had an inadequate response to anakinra, so any indirect comparison between the agents is affected by selection bias and should be interpreted with caution. In the review of treatment with IL-1 inhibitors by Junge and colleagues, efficacy outcomes were measured in the same ways across different reports [77]. According to this review, anakinra is characterized by rapid and often sustained efficacy, leading to a reduced need for corticosteroids. Systemic symptoms resolve quickly, but joint symptoms may be more persistent [77]. Anakinra has a short half-life, so the efficacy is relatively short-lived and daily administration is required [13]. Besides ISRs that occur with daily injections, other adverse events associated with anakinra include infections, elevated liver enzymes, mild leukopenia, and myopathy [13].

The review by Junge and colleagues noted that canakinumab was effective in difficult-to-treat AOSD patients and was well tolerated [77]. To date, the published reports on the use of canakinumab in AOSD describe its use in patients previously treated with other anti-IL1 agents, mainly anakinra. These reports demonstrate that canakinumab is effective even in these difficult-to-treat patients who are likely to have more severe disease. Based on our systematic review of the literature and on the most recent review by Galozzi and colleagues [20], there are only 24 published cases of patients with AOSD who were treated with canakinumab. These reports show efficacy in reducing both systemic and articular manifestations, while reducing the need for corticosteroids. These preliminary findings of efficacy are being confirmed in a multicenter, randomized placebo-controlled trial with canakinumab in AOSD patients with active joint involvement (NCT02204293) that is currently underway in Germany [142].

### Early versus late treatment of AOSD with IL-1 inhibitors

Statement 4.1. There are no available studies comparing the efficacy of treatment with IL-1 inhibitors in early versus late stages of AOSD. Data from SJIA suggest that early treatment with IL-1 inhibitors is associated with a better therapeutic response [5]. [Grade D]

Statement 4.2. Indirect data show that IL-1 inhibitors can be effective in AOSD, both in first and subsequent lines of biologic treatment [2b]. [Grade B]

Early use (soon after disease onset) of IL-1 inhibitors in AOSD has not been explored yet. Direct comparisons of early versus late initiation of treatment with IL-1 inhibitors are also not available. The evidence from studies in SJIA suggests that early treatment with anti-IL-1 therapy is associated with a better response and that IL-1 inhibitors may be a valuable option in the first-line disease setting [77, 143–145]. Indirect evidence from the available reports suggests that IL-1β blockade is effective as the first-line intervention, as well as in later lines of treatment (up to the sixth line of treatment with biologics) [8, 57–60, 62, 64, 68, 70].

### Efficacy of treatment therapy with anti-IL-1 agents on systemic and joint disease

Statement 5.1. Some data suggest that IL-1 inhibitors may be more effective on systemic rather than chronic articular manifestations of AOSD [2b]. [Grade C]

To date, there is no study specifically aimed at comparing the effects of anti-IL-1 therapy on systemic versus articular manifestations of AOSD. However, indirect evidence suggests that anti-IL-1 agents may be more effective at relieving systemic disease [6, 8, 60, 62, 66, 68, 70, 77]. Specifically, in the study by Giampietro et al., a complete response to anakinra was reported in 46.7% of patients with systemic disease (*n* = 15) compared to 38.4% of patients with the chronic articular pattern (*n* = 13) [66]. A much higher difference in terms of response was reported by Cavalli et al. who identified a complete rate of response in 91.6% of patients with systemic disease (*n* = 12) compared to 37.5% of patients with articular disease (*n* = 8) [70]. These findings are consistent with the pattern of response reported in children with SJIA, in whom IL-1 inhibitors appear to be more effective in improving non-articular signs and symptoms (such as fever, rash, and inflammatory markers) than on the arthritic features of the disease [60, 146–148].

## Discussion

A large body of evidence supports the similarity between AOSD and SJIA. Furthermore, both conditions respond to agents that inhibit IL-1β by distinct mechanisms, which confirm the pivotal role of IL-1β in the pathogenesis of these conditions.

The likely differences in pharmacokinetics and pharmacogenetics between AOSD and SJIA do not allow us to extrapolate information on the effectiveness and safety profile of IL-1 inhibitors from SJIA to AOSD. This is the main reason why we decided to perform this systematic review of the literature specifically on AOSD. The aim of this study was to clearly collect and summarize all of the available evidence supporting the use of IL-1 inhibitors specifically in patients with AOSD, for which information is scarce compared with SJIA. While studies on the pharmacokinetics of anakinra [149] or canakinumab [150] in patients with SJIA are available, there are still no comparable data in patients with AOSD. Nonetheless, there should be obvious differences in IL-1 inhibitor pharmacokinetics related to age, body weight, and genetic profile. This crucial point underlines the need to summarize current evidence on AOSD in order to provide guidelines for clinicians prescribing these drugs in this rare condition.

Most of the data on the efficacy and safety of IL-1 blockers in AOSD describe the use of anakinra, and the evidence from RCTs is limited to one small study investigating this medication [18]. Nevertheless, the available data consistently demonstrate a clinical benefit with these agents, with only a few patients being non-responsive to IL-1 blockers. Some reports describe patients who experience rapid and marked improvement in the signs and symptoms of AOSD after the first injection [6, 59, 62, 99, 100]. In patients who respond to IL-1 inhibitor therapy, complete remission can be maintained for prolonged periods (≥ 12 months) [59, 80, 87, 96, 100, 103]. Data from case reports provide anecdotal support for the use of a different IL-1 inhibitor in patients who do not respond or only partially respond to the first agent [80–83, 85, 87].

We used a modified Delphi process to develop the reported consensus statements. This technique has the advantage of being able to systematically synthesize the knowledge and opinions of a large group of individuals with diverse expertise and from different geographic locations, thereby limiting the potential for a small group of experts from one area to dominate [25] (Appendix). By collecting responses anonymously, the Delphi method is able to counteract the effect of any psychological pressures that can influence opinions in a small-group discussion environment, such as ideological or status differences or a tendency by some members to dominate others. When conducted well, the Delphi method can provide valid guidance to clinical practice in situations where there is limited empirical evidence [25]. Our process was robust, the participation rate by Italian physicians with expertise in auto-inflammatory conditions was high, and our panel included a nationally representative sample of experts from most geographic regions in Italy (Table 1). Moreover, there was a high rate of consensus on most statements. These results reflect the favorable outcomes of clinical reports on the use of IL-1 inhibitors in patients with AOSD, as well as the physicians’ own positive clinical experience with these agents.

Our study is not without limitations. We set a relatively low threshold for consensus (> 66%) [151], although all of the statements generated consensus of ≥ 88%, so the chosen threshold does not affect the validity of the consensus. Delphi-based research is a method for *making the best use of available information* [24]. However, it should not replace properly conducted scientific research, and it is important to emphasize that the current recommendations should be validated in well-designed, prospective clinical studies. Indeed, it should be taken into account that data coming from our systematic review of the literature are mainly based on retrospective study cohorts. Data on anakinra include one RCT [18], while data on canakinumab come only from small case series and a single pooled analysis of study data [33, 76, 77]. In addition, the majority of data on canakinumab come from AOSD patients previously treated with anakinra, with a consequent selection bias for patients with the most aggressive and difficult to treat forms of AOSD [76, 77]. Thus, more data on this therapy are needed in patients with AOSD, particularly on its use as the first-line IL-1 inhibitor. Of note, at the time when we performed the systematic review of the literature, there were no studies evaluating the efficacy of canakinumab in AOSD as the first-line therapy. A study by Cavalli and colleagues, which evaluated the effectiveness of canakinumab as the first-line biologic agent in patients with AOSD and reported excellent clinical response rates, has since been published [152].

Although the evidence of the overall efficacy of anti-IL1 agents in patients with AOSD, especially in those with the systemic form of the disease, is quite convincing, the effectiveness of these medications on articular involvement should be further explored. For this reason, the results of the ongoing RCT with canakinumab (NCT02204293) in patients with AOSD and active joint involvement are eagerly awaited.

Data on anakinra in patients with AOSD are mainly based on retrospective observational studies. Following the only existing RCT of anakinra in patients with AOSD, the results of which were published in 2012 [18]; a new RCT on anakinra in AOSD is currently ongoing (NCT03265132). This trial aims to evaluate anakinra efficacy in newly diagnosed SJIA and AOSD patients and to investigate its safety, pharmacokinetics (PK), and immunogenicity. The data from this trial, which is enrolling patients with a disease duration of less than 6 months, will provide key information regarding the efficacy of this treatment, especially in the early disease stages.

## Conclusions

Although adequately powered RCTs on the use of IL-1 inhibitors in AOSD are lacking, the literature is consistent in demonstrating a beneficial effect of these agents, with a high proportion of patients achieving rapid and sustained remission of systemic symptoms and normalization of inflammatory markers. Given the absence of a strong evidence base for the use of IL-1 inhibitors in AOSD, a robust and careful Delphi process was undertaken to develop consensus recommendations on the use of these agents, which we hope will assist physicians in the rational prescribing of these agents for patients affected by this condition.

### Supplementary information

**Additional file 1: Table S1**. Search strategy^†^ to identify articles describing similarities and differences between SJIA and AOSD. **Table S2**. Search strategy^†^ to identify articles describing the efficacy and safety of IL-1 blockade in AOSD. **Table S3**. Case reports (with ≤5 patients) describing the use of anti-IL-1 therapy in patients with AOSD.

## Data Availability

All data generated or analyzed during this study are included in this published article and its supplementary information files.

## Appendix

### Experts who contributed to the project by participating in the Delphi process

Stefano Alivernini, Institute of Rheumatology and Affine Sciences, Division of Rheumatology, Catholic University of the Sacred Heart, Rome

Elena Baldissera, Unit of Immunology, Rheumatology, Allergy and Rare Diseases (UnIRAR), IRCCS San Raffaele Scientific Institute, Milan

Elena Bartoloni, Rheumatology Unit, Department of Medicine, University of Perugia, Perugia

Alvise Berti, Department of Rheumatology, Santa Chiara Hospital, University of Trento, Trento

Serena Bugatti, Department of Rheumatology, IRCCS Policlinico San Matteo Foundation, University of Pavia, Pavia

Dario Camellino, Division of Rheumatology, La Colletta Hospital, Arenzano

Daniele Cammelli, Department of Experimental and Clinical Medicine, University of Florence, Florence

Roberto Caporali, Department of Rheumatology, IRCCS Policlinico San Matteo Foundation, University of Pavia, Pavia.

Francesco Caso, Rheumatology Unit, Department of Clinical Medicine and Surgery, University Federico II, Naples

Elena Cavallaro, Department of Medical and Biological Sciences, Rheumatology Clinic, University of Udine, Udine

Giulio Cavalli, Department of Internal Medicine, Vita-Salute San Raffaele University, Milan

Michele Colaci, Department of Clinical and Experimental Medicine, Internal Medicine Unit, Cannizzaro Hospital, University of Catania, Catania

Luisa Costa, Rheumatology Unit, Department of Clinical Medicine and Surgery, University Federico II, Naples

Gerardo di Scala, Rheumatology Section/Immunoallergology Unit, AOU Careggi, Florence

Giacomo Emmi, Rheumatology Section/Immunoallergology Unit, AOU Careggi, Florence

Micol Frassi, Rheumatology and Clinical Immunology, Spedali Civili and Department of Clinical and Experimental Sciences, University of Brescia, Brescia

Roberto Gerli, Rheumatology Unit, Department of Medicine, University of Perugia, Perugia

Roberto Giacomelli, Department of Biotechnological and Applied Clinical Science, Division of Rheumatology, University of L’Aquila, L’Aquila

Elisa Gremese, Institute of Rheumatology and Affine Sciences, Division of Rheumatology, Catholic University of the Sacred Heart, Rome

Florenzo Iannone, Rheumatology Unit, Interdisciplinary Department of Medicine, University of Bari, Bari

Giovanni Lapadula, Rheumatology Unit, Interdisciplinary Department of Medicine, University of Bari, Bari

Lorenzo Leveghi, Department of Rheumatology, Santa Chiara Hospital, University of Trento, Trento

Andrea Lo Monaco, Rheumatology Unit, Department of Clinical and Experimental Medicine, Sant’Anna Hospital, University of Ferrara, Ferrara

Giuseppe Lopalco, Rheumatology Unit, Interdisciplinary Department of Medicine, University of Bari, Bari

Raffaele Manna, Periodic Fever Research Center, Institute of Internal Medicine, Catholic University of the Sacred Heart, Fondazione Policlinico A. Gemelli, Rome

Daniela Marotto, Rheumatology, Department of Medical Sciences and Public Health, Assl Olbia, Olbia

Alessandro Mathieu, Rheumatology Unit, Department of Medical Sciences, University and AOU of Cagliari, Cagliari

Rossella Neri, Rheumatology Unit, Department of Clinical and Experimental Medicine, University of Pisa, Pisa

Isabella Patisso, Periodic Fever Research Center, Institute of Internal Medicine, Catholic University of the Sacred Heart, Fondazione Policlinico A. Gemelli, Rome

Matteo Piga, Rheumatology Unit, Department of Medical Sciences, University and AOU of Cagliari, Cagliari

Leonardo Punzi, Rheumatology Unit, Department of Medicine - DIMED, University of Padova, Padova

Micol Romano, Division of Rheumatology, ASST Gaetano Pini, Milan

Piero Ruscitti, Department of Biotechnological and Applied Clinical Science, Division of Rheumatology, University of L’Aquila, L’Aquila

Carlo Salvarani, Rheumatology Unit, Azienda USL-IRCCS di Reggio Emilia and University of Modena e Reggio Emilia

Raffaele Scarpa, Rheumatology Unit, Department of Clinical Medicine and Surgery, University Federico II, Naples

Rossana Scrivo, Rheumatology Unit, Department of Internal Medicine and Medical Specialties, Sapienza University of Rome, Rome

Rosaria Talarico, Rheumatology Unit, Department of Clinical and Experimental Medicine, University of Pisa, Pisa

Elena Verrecchia, Periodic Fever Research Center, Institute of Internal Medicine, Catholic University of the Sacred Heart, Fondazione Policlinico A. Gemelli, Rome

Ombretta Viapiana, Rheumatology Unit, Department of Medicine, University of Verona, Verona

Antonio Vitale, Research Center of Systemic Autoinflammatory Diseases and Behçet’s Disease, Clinic Surgery and Neurosciences, Department of Medical Sciences, Surgery and Neurosciences, University of Siena, Siena

Gianfranco Vitiello, Department of Experimental and Clinical Medicine, University of Florence, Florence

## Abbreviations

ACR: American College of Rheumatology
AOSD: Adult-onset Still’s disease
EULAR: European League Against Rheumatism
EMA: European Medicines Agency
EU: European Union
IL: Interleukin
ILAR: International League of Associations for Rheumatology
NSAIDs: Non-steroidal anti-inflammatory drugs
PICO: Patient, Intervention, Control, Outcomes
RCT: Randomized controlled trial
SIR: Società Italiana Reumatologia
SJIA: Systemic juvenile idiopathic arthritis
