# Correction to: Management of adult-onset Still’s disease with interleukin-1 inhibitors: evidence- and consensus-based statements by a panel of Italian experts

**DOI:** 10.1186/s13075-019-2094-5

**Published:** 2020-01-09

**Authors:** Serena Colafrancesco, Maria Manara, Alessandra Bortoluzzi, Teodora Serban, Gerolamo Bianchi, Luca Cantarini, Francesco Ciccia, Lorenzo Dagna, Marcello Govoni, Carlomaurizio Montecucco, Roberta Priori, Angelo Ravelli, Paolo Sfriso, Luigi Sinigaglia, AOSD Consensus Group, Stefano Alivernini, Elena Baldissera, Elena Bartoloni, Alvise Berti, Serena Bugatti, Dario Camellino, Daniele Cammelli, Roberto Caporali, Francesco Caso, Elena Cavallaro, Giulio Cavalli, Michele Colaci, Luisa Costa, Gerardo di Scala, Giacomo Emmi, Micol Frassi, Roberto Gerli, Roberto Giacomelli, Elisa Gremese, Florenzo Iannone, Giovanni Lapadula, Lorenzo Leveghi, Giuseppe Lopalco, Raffaele Manna, Daniela Marotto, Alessandro Mathieu, Rossella Neri, Isabella Patisso, Matteo Piga, Leonardo Punzi, Micol Romano, Piero Ruscitti, Carlo Salvarani, Raffaele Scarpa, Rossana Scrivo, Rosaria Talarico, Elena Verrecchia, Ombretta Viapiana, Antonio Vitale, Gianfranco Vitiello

**Affiliations:** 1 Dipartimento di Scienze Cliniche, Internistiche, Anestesiologiche e Cardiovascolari, Rheumatology Unit, Sapienza University of Rome, Rome, Italy; 2 Division of Rheumatology, ASST Gaetano Pini-CTO, Milan, Italy; 3 Rheumatology, Department of Medical Sciences, University of Ferrara and Azienda Ospedaliera-Universitaria di Ferrara, Cona, FE Italy; 4 SC Reumatologia, ASL3 - Azienda Sanitaria Genovese, Genoa, Italy; 5 Department of Medical Sciences, Surgery and Neurosciences, Rheumatology Unit, University of Siena, Policlinico “Le Scotte”, Siena, Italy; 6 Rheumatology, Dipartimento di Medicina di Precisione, Università della Campania “L. Vanvitelli”, Naples, Italy; 7 Unit of Immunology, Rheumatology, Allergy and Rare Diseases (UnIRAR), IRCCS San Raffaele Scientific Institute, Milan, Italy; 8 Department of Rheumatology, IRCCS Policlinico San Matteo Foundation, University of Pavia, Pavia, Italy; 9 Clinica Pediatrica e Reumatologia, Istituto Giannina Gaslini and Università degli Studi di Genova, Genoa, Italy; 10 Rheumatology Unit, Department of Medicine, University of Padua, Padua, Italy

**Correction to: Arthritis Res Ther (2019) 21:275**

**https://doi.org/10.1186/s13075-019-2021-9**

Following publication of the original article [1], it was brought to our attention that the AOSD Consensus Group was incorrectly tagged and therefore not searchable. The publishers apologize for this error.
