# Supplementary material for: Management of adult-onset Still’s disease with interleukin-1 inhibitors: evidence- and consensus-based statements by a panel of Italian experts
Source: Arthritis Res Ther. 2019 Dec 11;21:275. doi: 10.1186/s13075-019-2021-9 (PMC6907145; doi:10.1186/s13075-019-2021-9)
Supplement: Supplementary file 1 — Additional file 1: Table S1. Search strategy† to identify articles describing similarities and differences between SJIA and AOSD. Table S2. Search strategy† to identify articles describing the efficacy and safety of IL-1 blockade in AOSD. Table S3. Case reports (with ≤5 patients) describing the use of anti-IL-1 therapy in patients with AOSD. [file 13075_2019_2021_MOESM1_ESM.docx]

# Supplementary Materials

**Management of adult-onset Still’s disease with interleukin-1 inhibitors: evidence- and consensus-based statements by a panel of Italian experts**

***Arthritis Research & Therapy***

**Authors:** *Serena Colafrancesco, ^*^Maria Manara, Alessandra Bortoluzzi, Teodora Serban, Gerolamo Bianchi, Luca Cantarini, Francesco Ciccia, Lorenzo Dagna, Marcello Govoni, Carlomaurizio Montecucco, Roberta Priori, Angelo Ravelli, Paolo Sfriso^1^, Luigi Sinigaglia

*These authors share first authorship.

**Corresponding author:** Serena Colafrancesco, Department of Internal Medicine and Medical Specialties, Rheumatology Unit, Sapienza University of Rome, Rome, Italy; e-mail: [serena.colafrancesco18@gmail.com](mailto:serena.colafrancesco18@gmail.com)

## Table S1. Search strategy^†^ to identify articles describing similarities and differences between SJIA and AOSD.

| **Step** | **Search Terms** | **Results** |
| --- | --- | --- |
| 1 | (Systematic Juvenile idiopathic Arthritis or SJIA or Systemic juvenile rheumatoid arthritis or Systematic-onset Juvenile Idiopathic Arthritis or Systematic Onset Juvenile Idiopathic Arthritis or Systemic-onset juvenile rheumatoid arthritis or Systemic onset juvenile rheumatoid arthritis or Systemic-onset juvenile arthritis or Systemic juvenile arthritis or Systemic juvenile idiopathic arthritis or Systemic onset juvenile idiopathic arthritis or Systemic-onset juvenile idiopathic arthritis or Systemic juvenile rheumatoid arthritis or Systemic onset juvenile rheumatoid arthritis or Systemic-onset juvenile rheumatoid arthritis or child* or juvenile or adolescen* or child* still* or Juvenile-onset still* or Juvenile onset still* or Childhood onset still* or Childhood-onset still* or Child onset stills or Child-onset still* or (still* disease and child*)).ti,ab. | 3820051 |
| 2 | (Adult Onset Still* Disease or Adult-Onset Still* Disease or AOSD or Adult Still* Disease or (adult and still* disease)).ti,ab. | 4332 |
| 3 | 1 and 2 | 532 |
| 4 | Remove duplicates from 3 | 332 |

AOSD, adult-onset Still’s disease; SJIA, systemic juvenile idiopathic arthritis.

^†^The following databases were searched: Biosis Previews (1995 to 2018 Week 21), Embase (1974 to 2018 April 20), Medline (1946 to 2018 April 20).

## Table S2. Search strategy^†^ to identify articles describing the efficacy and safety of IL-1 blockade in AOSD.

| **Step** | **Search Terms** | **Results** |
| --- | --- | --- |
| 1 | (Adult Onset Still* Disease or Adult-Onset Still* Disease or AOSD or Still* Disease or ASD or Still8 syndrome).ti,ab. | 59282 |
| 2 | ((IL1 adj (inhibit* or block* or antagon*)) or (IL-1 adj (inhibit* or block* or antagon*)) or (IL 1 adj (inhibit* or block* or antagon*)) or (interleukin1 adj (inhibit* or block* or antagon*)) or (interleukin-1 adj (inhibit* or block* or antagon*)) or (interleukin 1 adj (inhibit* or block* or antagon*)) or ((inhibit* or block* or antagon*) adj IL1) or ((inhibit* or block* or antagon) adj IL-1) or ((inhibit* or block or antagon*) adj IL 1) or ((inhibit* or block* or antagon*) adj interleukin1) or ((inhibit* or block* or antagon*) adj interleukin 1) or ((inhibit* or block* or antagon*) adj interleukin-1)).ti,ab. | 7874 |
| 3 | ((IL1 receptor adj (inhibit* or block* or antagon*)) or (IL-1 adj (inhibit* or block* or antagon*)) or (Il 1 receptor adj (inhibit* or block* or antagon*)) or ( interleukin1 receptor adj (inhibit* or block* or antagon*)) or (interleukin-1 receptor adj (inhibit* or block* or antagon*)) or (interleukin 1 receptor adj (inhibit* or block* or antagon*)) or ((inhibit* or block* or antagon*) adj IL1) or ((inhibit* or block* or antagon*) adj IL-1) or ((inhibit* or block* or antagon*) adj IL 1) or ((inhibit* or block* or antagon*) adj interleukin1) or ((inhibit* or block* or antagon*) adj interleukin-1) or ((inhibit* or block* or antagon*) adj interleukin 1)).ti,ab. | 22263 |
| 4 | ((IL1b adj ((inhibit* or block* or antagon*)) or (IL-1b adj (inhibit* or block* or antagon*)) or (IL 1b adj (inhibit* or block* or antagon*) or ( interleukin1b adj (inhibit* or block* or antagon*)) or (interleukin-1b adj (inhibit* or block* or antagon*)) or (interleukin 1b adj (inhibit* or block* or antagon*)) or (IL1b receptor adj (IL-1b receptor adj (inhibit* or block* or antagon*)) or IL-1b receptor adj (IL-1b adj (inhibit* or block* or antagon*)) or interleukin1b receptor adj (IL-1b adj (inhibit* or block* or antagon*)) or (interleukin-1b receptor adj (inhibit* or block* or antagon*)) or (interleukin 1b receptor adj (inhibit* or block* or antagon*)) or ((inhibit* or block* or antagon*) adj IL1b) or ((inhibit* or block* or antagon*) adj IL-1b) or ((inhibit* or block* or antagon*) adj IL 1b) or ((inhibit* or block* or antagon*) adj interleukin1b) or ((inhibit* or block* or antagon*) adj interleukin 1b) or ((inhibit* or block* or antagon*) adj interleukin-1b)).ti,ab. | 176 |
| 5 | ((IL1 beta adj (inhib(inhibit* or block* or antagon*)it* or block* or antagon*)) or (IL-1 beta adj (inhibit* or block* or antagon*)) or (IL 1 beta adj (inhibit* or block* or antagon*)) or (interleukin1 beta adj (inhibit* or block* or antagon*) or (interleukin-1 beta adj (inhibit* or block* or antagon*)) or (interleukin 1 beta adj (inhibit* or block* or antagon*)) or (IL1 beta receptor adj (inhibit* or block* or antagon*)) or (IL-1 beta receptor adj (inhibit* or block* or antagon*)) or (IL 1 beta receptor adj (inhibit* or block* or antagon*)) or (interleukin1 beta receptor adj (inhibit* or block* or antagon*)) or (interleukin-1 beta receptor adj (inhibit* or block* or antagon*)) or (interleukin 1 beta receptor adj (inhibit* or block* or antagon*)) or ((inhibit* or block* or antagon*) adj IL1 beta) or ((inhibit* or block* or antagon*) adj IL-1 beta) or ((inhibit* or block* or antagon*) adj IL 1 beta) or ((inhibit* or block* or antagon*) adj interlaukin1 beta) or ((inhibit* or block* or antagon*) adj interleukin 1 beta) or ((inhibit* or block* or antagon*) adj interleukin-1 beta)).ti,ab. | 1983 |
| 6 | ((Anti-IL1 adj (treat* or therap* or medic*)) or (Anti IL1 adj (treat* or therap* or medic*)) or (Anti-IL-1 adj (treat* or therap* or medic*)) or (Anti IL-1 adj (treat* or therap* or medic*)) or (Anti-IL 1 adj (treat* or therap* or medic*)) or (Anti IL 1 adj (treat* or therap* or medic*)) or (Anti-interleukin1 adj (treat* or therap* or medic*)) or (Anti interleukin1 adj (treat* or therap* or medic*)) or (Anti-interleukin-1 adj (treat* or therap* or medic*)) or (Anti interleukin-1 adj (treat* or therap* or medic*)) or (Anti-interleukin 1 adj (treat* or therap* or medic*)) or (Anti interleukin 1 adj (treat* or therap* or medic*)) or ((treat* or therap* or medic*) adj Anti-IL1) or ((treat* or therap* or medic*) adj Anti IL1) or ((treat* or therap* or medic*) adj Anti-IL-1) or ((treat* or therap* or medic*) adj Anti IL-1) or ((treat* or therap* or medic*) adj Anti-IL 1) or ((treat* or therap* or medic*) adj Anti IL 1) or ((treat* or therap* or medic*) adj Anti-interleukin1) or ((treat* or therap* or medic*) adj Anti interleukin1) or ((treat* or therap* or medic*) adj Anti-interleukin-1) or ((treat* or therap* or medic*) adj Anti interleukin-1) or ((treat* or therap* or medic*) adj Antiinterleukin 1) or ((treat* or therap* or medic*) adj Anti interleukin 1)).ti,ab. | 468 |
| 7 | (Anakinra or Antrilor or Kineret or L-04AC03 or L04AC03 or Anakinrum).ti,ab. or 143090-92-0.rn. | 8566 |
| 8 | (rilonacept or Arcalyst or RGN-303* or RGN 303* or RGN303*).ti,ab. or 501081-76-1.rn. | 997 |
| 9 | (ACZ-885* or ACZ885* or ACZ 885*or canakinumab* or ilaris*).ti,ab. or (402710-25-2 or 402710-27-4 or 914613-48-2).rn. | 2825 |
| 10 | 2 or 3 or 4 or 5 or 6 or 7 or 8 or 9 | 33146 |
| 11 | 1 and 10 | 542 |
| 12 | Remove duplicates from 11 | 358 |

AOSD, adult-onset Still’s disease; IL, interleukin.

^†^The following databases were searched: Biosis Previews (1995 to 2018 Week 21), Embase (1974 to 2018 April 20), Medline (1946 to 2018 April 20).

## Table S3. Case reports (with ≤5 patients) describing the use of anti-IL-1 therapy in patients with AOSD.

| **Case reports of IL-1 inhibitor efficacy** | | | | |
| --- | --- | --- | --- | --- |
| **Reference** | **N** | **Patient(s)** | **Treatment** | **Effect** |
| Rudinskaya and Trock 2003 [[1](#_ENREF_1)] | 1 | 37yo male with severe AOSD refractory to high-dose CS + MTX | Ankinra 100 mg/day + CS + MTX | Rapid improvement in clinical symptoms and laboratory markers of disease activity; CS tapered and discontinued and MTX dose reduced without relapse. The patient remained in remission over 12 months of follow-up. |
| Vasques Godinho et al. 2005 [[2](#_ENREF_2)] | 1 | 32yo female with referactory AOSD despite treatment with MTX, CS, naproxen, IVIg and anti-TNF agents (infliximab and etanercept) | Anakinra 100 mg/day + CS + MTX + naproxen | Rapid clinical improvement and normalization of laboratory features leading to sustained clinical remission + discontinuation of CS and naproxen (18 months of follow-up) |
| Goldbach-Mansky et al. 2006 [[3](#_ENREF_3)] | 2 | 2 patients with AOSD and other auto-inflammatory conditions | Rilonacept 100 mg/day for 3 doses, 7 days off, then for 1 year if responding | Patient A: Clinical and laboratory improvement during treatment followed by flare when rilonacept withdrawn; remission maintained on 100 mg/week  Patient B: Clinical improvement, but laboratory parameters remained elevated throughout despite rilonacept 360 mg/week. |
| Kötter et al. 2007 [[4](#_ENREF_4)] | 4 | 27yo female with repeated flares despite CS, DMARDs, MTX, and TNF-α blockers; 40yo male intolerant of DMARDs and MTX with flares on CS; 33yo female refractory to CS + MTX; 28yo female symptomatic during CS + MTX | Anakinra | Improvement in signs and symptoms within 1–7 days of starting anakinra; sustained remission for 12–15 months, and CS tapered in all patients |
| Mubashir et al. 2007 [[5](#_ENREF_5)] | 1 | 29yo female with AOSD who had received CS, DMARDs, NSAIDs, MTX and sulfasalazine; developed pulmonary hypertension | Anakinra mg/day + CS + nifedipine | Improvement in systemic and articular AOSD, but experienced progressive heart failure and died |
| Debiais et al. 2008 [[6](#_ENREF_6)] | 1 | 33yo patient with AOSD refractory to MTX, CS, IVIg, infliximab, etanercept, and cyclosporine A | Anakinra100 mg/day + MTX | Symptoms resolved within 48 hours; patient remained asymptomatic for ≥7 months and CS could be tapered. |
| Gesierich et al. 2008 [[7](#_ENREF_7)] | 1 | 50yo female with AOSD receiving CS | Anakinra 100 mg/day + MTX | Signs and symptoms of AOSD resolved, CS dose was tapered and patient asymptomatic for ≥10 months |
| Mylona et al. 2008 [[8](#_ENREF_8)] | 1 | 46yo male with AOSD receiving CS who developed hepatic dysfunction | Anakinra 100 mg/day + CS | Systemic features improved in first weeks of treatment; even with CS tapering, liver function normalized by 5 months and pt was in full remission |
| Priori et al. 2008 [[9](#_ENREF_9)] | 3 | 22-32yo females with AOSD and severe joint involvement that was resistant to conventional treatment, including CS, MTX and hydroxychloroquine | Anakinra 100 mg/day + CS | Improvement in joint involvement in all patients, with ultrasound showing a good response to treatment at 3 months; CS could be tapered in all patients. Two patients received long-term anakinra, with one still in remission after 29 months and the other in remission up to 12 months, when a flare was treated with increased CS dose. In the remaining patient, anakinra was discontinued after 8 weeks because of an adverse event (diffuse itching rash), but remission was maintained with MTX (previously ineffective) and low-dose CS during 6 months of follow-up. |
| De Bandt and Saint-Marcoux 2009 [[10](#_ENREF_10)] | 1 | 34yo female with AOSD refractory to multiple drugs, including CS, MTX, leflunomide, thalidomide, etanercept and infliximab | Anakinra 100 mg/day | No response to anakinra, but clinical remission and discontinuation of CS and MTX during subsequent treatment with tocilizumab |
| El Karoui et al. 2009 [[11](#_ENREF_11)] | 1 | 36yo female with AOSD, complicated by thrombotic microangiopathy, Purtcher-like retinopathy and lower extremity gangrene | Anakinra 100 mg/day + CS | Gradual resolution of systemic and articular disease activity over 7 months with CS tapering; incomplete improvement in visual acuity and bilateral amputation of forefeet still required |
| Sentić et al. 2009 [[12](#_ENREF_12)] | 1 | 32yo female with refractory AOSD despite treatment with high-dose CS, MTX, IVIg and NSAID | Anakinra + low-dose CS | Rapid improvement in clinical condition and acute phase reactants |
| Lahiri and Teng 2010 [[13](#_ENREF_13)] | 1 | 40yo female with AOSD who was intolerant of CS and infliximab, and relapsed with DMARD + MTX | Anakinra 100 mg/day + MTX + low-dose CS | Rapid improvement and sustained remission for ≥12 months with alternate day dosing; CS doses were tapered without relapse |
| Moulis et al. 2010 [[14](#_ENREF_14)] | 2 | 42yo male with AOSD with chorioretinopathy on CS; 18yo female with AOSD unresponsive to CS | Anakinra 100 mg/day | Rapid improvement in symptoms in both patients; complete remission maintained despite CS discontinuation and anakinra tapering (male) or withdrawal (female) |
| Perdan-Pirkmajer et al. 2010 [[15](#_ENREF_15)] | 1 | 35yo male with AOSD | Anakinra 100 mg/day | Excellent clinical and laboratory improvement followed by disease flare at 2 months; MTX and CS added without success so anakinra discontinued; the patient eventually responded to tocilizumab after lack of response to of etanercept |
| Quartuccio et al. 2010 [[16](#_ENREF_16)] | 1 | 57yo male with AOSD refractory to CS, MTX and adalimumab | Anakinra 100 mg/day + CS | Rapid clinical improvement, but anakinra had to be discontinued because of anakinra-related thrombocytopenia at 2 weeks; the patient eventually responded to abatacept after lack of response to anti-TNF agents (etanercept and infliximab) |
| Tamaki et al. 2010 [[17](#_ENREF_17)] | 1 | 23yo female with refractory AOSD despite high-dose CS, IVIg and MTX | Anakinra 100 mg/day + CS + MTX | Rapid remission, and CS successfully tapered without exacerbation of symptoms or laboratory parameters |
| Agarwal et al. 2011 [[18](#_ENREF_18)] | 1 | 21yo male with AOSD developed MAS and disseminated histoplasmosis during treatment with adalimumab | Anakinra 100 mg/day + high-dose CS | Recovery |
| Brusch et al. 2011 [[19](#_ENREF_19)] | 1 | Male in early 50s with refractory AOSD previously treated with CS, MTX, leflunomide | Anakinra 100 mg/day | Immediate resolution of symptoms and normalization of inflammatory markers; recurrence of symptoms when anakinra temporarily unavailable but otherwise symptom-free over 6 months of follow-up |
| Fischer-Betz et al. 2011 [[20](#_ENREF_20)] | 2 | 27yo and 29yo pregnant females with AOSD | Anakinra 100 mg/day + CS | Rapid induction/maintenance of remission; CS-sparing effect; no adverse developmental effects in utero or postpartum (anakinra taken throughout pregnancy in one patient and initiated after the first trimester in the other) |
| Raffeiner et al. 2011 [[21](#_ENREF_21)] | 1 | 20yo male with AOSD presenting with myocarditis | Anakinra 100 mg/day + CS | Rapid clinical improvement and complete resolution of myocarditis; CS tapered and stopped at 3 months and anakinra continued for 12 months; still in complete remission at 22 months of follow-up |
| Rech et al. 2011 [[22](#_ENREF_22)] | 3 | Two 19yo females and a 73yo male with refractory AOSD despite CS treatment | Anakinra +CS ± MTX | No to slight improvement in symptoms and acute phase reactants remained high during anakinra treatment (anti-TNF agents also used unsuccessfully in 2 patients), but complete remission was subsequently achieved in all patients after initiation of tocilizumab |
| Jaqua et al. 2012 [[23](#_ENREF_23)] | 1 | 26yo male with surgical asplenia who was admitted to PCU with SIRS before AOSD diagnosis | CS + anakinra | Good response to anakinra allowing tapering of CS |
| Kontzias et al. 2012 [[24](#_ENREF_24)] | 2 | 38yo male with flares during anakinra + CS treatment; 36yo female who relapsed after anakinra + CS then rilonacept + CS | Canakinumab | Sustained remission achieved with canakinumab in both, with marked steroid tapering in male patient |
| Loh et al. 2012 [[25](#_ENREF_25)] | 1 | 20yo male with AOSD and MAS uncontrolled by CS + cyclosporine | Anakinra 200 mg then 100 mg/day | Complete remission maintained; CS and cyclosporine withdrawn and replaced with MTX |
| Petryna et al. 2012 [[26](#_ENREF_26)] | 3 | 41yo, 36yo and 44yo females with AOSD who were refractory or partially responsive to anakinra + CS and other therapies (DMARDs, MTX, azathioprine, abatacept) | Rilonacept | Complete and sustained remission (≥16–26 months); MTX and CS able to be tapers or withdrawn |
| Rech et al. 2012 [[27](#_ENREF_27)] | 1 | 47yo male with AOSD and chronic recurrent multifocal osteomyelitis previously treated with CS and ibuprofen | Anakinra 100 mg/day | Rapid clinical improvement and normalization of inflammatory parameters, with almost complete resolution of inflammatory foci on bone scan at 10 months, and continuation of anakinra (14 months of follow-up) |
| Eriksson et al. 2013 [[28](#_ENREF_28)] | 1 | 32yo male with AOSD phenotype but CAPS genotype who was intolerant of anakinra and refractory to etanercept and tocilizumab | Canakinumab 150 mg every 8 weeks | Rapid improvement in all joint and skin signs and symptoms, and normalization of laboratory parameters |
| Fischbach et al. 2013 [[29](#_ENREF_29)] | 1 | 21yo female presenting with pseudo-septic shock and acute respiratory distress syndrome associated with MAS after being treated with rituximab + CS for AOSD refractory to conventional therapy (MTX, azathioprine and hydroxychloroquine) | Anakinra 100 mg/day + CS | Resolution of rituximab-associated MAS and multi-organ dysfunction; CS could be discontinued |
| Orr et al. 2013 [[30](#_ENREF_30)] | 1 | 32yo male with AOSD diagnosed after liver transplant for acute liver failure caused by MAS | Anakinra + CS (previously maintained in remission for 6 years after liver transplant on cyclosporine, mycophenolate mofetil and CS until arthralgia, fever and rash provided evidence for an AOSD diagnosis) | Resolution of symptoms and normalization of graft function and serum ferritin, with the patient remaining well during 7 months of follow-up |
| Athanassiou et al. 2014 [[31](#_ENREF_31)] | 1 | 30yo female who was refractory to CS and anakinra 100 mg/day | Canakinumab | Complete remission; CS able to be tapered |
| Barsotti et al. 2014 [[32](#_ENREF_32)] | 1 | 30 yo female with AOSD, refractory to CS + MTX, intolerant of adalimumab, and with relapse after 2 years of successful treatment with anakinra | Canakinumab 150 mg every 4–8 weeks | Complete remission sustained for ≥18 months |
| Choi et al. 2014 [[33](#_ENREF_33)] | 1 | 37yo male; myocarditis was presenting sign at AOSD diagnosis | Anakinra 100 mg/day + CS | Complete clinical response; some myocardial fibrosis apparent on CT scan at 12 months  All treatment withdrawn by 12 months |
| Gutmark et al. 2014 [[34](#_ENREF_34)] | 1 | 23yo male; trochleitis was unusual presenting sign of AOSD | CS + anakinra | Systemic and ocular symptoms improved within 1 day of starting anakinra; CS were tapered after 3 months without symptom recurrence |
| Hartig et al. 2014 [[35](#_ENREF_35)] | 1 | 53yo male; NUD was unusual presenting sign of AOSD | Anakinra 100 mg/day | Skin lesions improved within 1 day of starting anakinra, and all symptoms had resolved within 1 week; patient remained symptom-free for >1 year |
| Lo Gullo et al. 2014 [[36](#_ENREF_36)] | 1 | 44yo female with AOSD refractory to, or intolerant of, DMARDs, cyclosporine A, anakinra, tocilizumab and infliximab | Canakinumab 150 mg every 8 weeks + MTX + CS | Resolution of systemic symptoms and improvement in articular disease, but with flares and persistent arthritis requiring CS |
| Yilmaz et al. 2014 [[37](#_ENREF_37)] | 1 | 28yo male with AOSD reactivation (7 years after liver transplant due to AOSD) while receiving mycophenolate mofetil and tacrolimus; inadequate response to initial treatment with high-dose CS + hydroxychloroquine + MTX | Anakinra 100 mg/day + CS | Rapid resolution of symptoms and normalization of laboratory parameters; mycophenolate mofetil discontinued 1 week after starting anakinra, and CS tapered and then discontinued at 2 months; the patient remained in complete remission during long-term follow-up (2.5 years) |
| Luconi et al. 2015 [[38](#_ENREF_38)] | 1 | 17yo male with AOSD and myocarditis unresponsive to CS + MTX | Anakinra 100 mg/day | Rapid improvement in clinical signs and symptoms and normalization of cardiac function; CS could be tapered within a few months |
| Michailidou et al. 2015 [[39](#_ENREF_39)] | 1 | 31yo male with AOSD | Anakinra 100 mg/day + CS | Systemic symptoms resolved quickly, but patient developed new rash that may have been due to AOSD or to anakinra |
| Waghmare et al. 2015 [[40](#_ENREF_40)] | 1 | 26yo male with AOSD presenting with myopericarditis and pleuritis, and refractory to tocilizumab + CS | Anakinra 100 mg/day + MTX + CS | Rapid clinical improvement, normalization of blood parameters and resolution of myopericarditis; CS could be gradually tapered; the patient remained stable after 18 months of follow-up |
| Agnihotri et al. 2016 [[41](#_ENREF_41)] | 1 | 28yo female; MAS was presenting sign at AOSD diagnosis | Anakinra 100 mg/day + CS | Recovery |
| Kumar et al. 2016 [[42](#_ENREF_42)] | 1 | 34yo male; MAS was presenting sign at AOSD diagnosis | Anakinra + CS + MTX | Patient self-discontinued anakinra because he disliked injections, and developed arthritis flares. MTX was replaced with mycophenolate mofetil. |
| Mehta et al. 2016 [[43](#_ENREF_43)] | 1 | 30yo female with previously undiagnosed and treated with IVIg AOSD presenting with pulmonary hypertension and MAS | Anakinra 100 mg twice daily + CS + cyclosporine | Resolution of pulmonary hypertension and MAS, with normalization of liver enzymes, cell counts, ferritin and inflammatory markers; pulmonary arterial pressure stable after 1 month of follow-up |
| Minori et al. 2016 [[44](#_ENREF_44)] | 1 | 59yo male with AOSD | Anakinra + CS | Significant clinical improvement with restoration of mobility and independence |
| Nataraja and Griffiths 2016 [[45](#_ENREF_45)] | 1 | 24yo female with AOSD and atypical pruritic rash unresponsive to CS and DMARDs | Anakinra 100 mg/day | Notable clinical and laboratory improvement within 1 month of starting anakinra |
| Yuhua et al. 2016 [[46](#_ENREF_46)] | 1 | 38yo female with AOSD refractory to CS, conventional DMARDs and anti-TNF agents (adalimumab and etanercept) | Anakinra 100 mg/day + CS + MTX | Rapid resolution of symptoms leading to complete remission; CS and MTX were discontinued and the anakinra dose reduced to 100 mg/20days |
| Parisi et al. 2017 [[47](#_ENREF_47)] | 1 | 42yo female with AOSD with MAS, disseminated intravascular coagulopathy, myocarditis and cardiac arrest | Anakinra 100 mg every 6 hours + IVIg + CS | Improvement in clinical and laboratory parameters, but recovery complicated by UTI and pancytopenia leading to anakinra dose reduction and withdrawal. After recovery of acute episode, patient maintained in AOSD remission on anakinra + cyclosporine |
| Albersmeyer et al. 2018 [[48](#_ENREF_48)] | 1 | 22 yo female with cystic fibrosis who was diagnosed with AOSD | Anakinra + CS | Rapid improvement in signs and symptoms. CS tapered and anakinra discontinued after 1 month with no recurrence of AOSD symptoms in the following 3 months |
| Breillat et al. 2018 [[49](#_ENREF_49)] | 1 | 45yo female with AOSD who was intolerant of tocilizumab and refractory to anakinra | Canakinumab 150 mg/month + CS | Sustained improvement in all symptoms and resolution of laboratory abnormalities; CS dose able to be tapered |
| Farrok Sheikh et al. 2018 [[50](#_ENREF_50)] | 1 | 56yo female with AOSD who had relapsed after discontinuing steroids after mania development | Anakinra 100 mg/day | Rapid clinical improvement in signs and symptoms of AOSD, and maintained clinical remission for ≥12 months |
| Piel-Julian et al. 2018 [[51](#_ENREF_51)] | 1 | 37yo male with AOSD complicated by acute myocarditis and initially treated with CS with no effect | Anakinra 100 mg/day + CS | Clinical symptoms fully resolved within 24 hours of initiating anakinra; CS were withdrawn after 1 month; anakinra dose decreased to 100 mg every other day at 6 months and withdrawn at 11 months after the patient developed acute respiratory failure related to staphylococcal pneumonia (the patient recovered and remained in complete remission 2 months later) |
| **Case reports of IL-1 inhibitor adverse events** | | | | |
| **Reference** | **N** | **Patient(s)** | **Treatment** | **Adverse event and outcome** |
| Guignard et al. 2007 [[52](#_ENREF_52)] | 1 | 23yo male with AOSD who relapsed after treatment (1) CS + MTX, (2) infliximab | Anakinra 100 mg/day | ARDS and SIRS developed 10 days after starting anakinra  Required ICU admission  Recovery except for surgical treatment of toe ischaemia  AOSD remission achieved on CS |
| Aly et al. 2013 [[53](#_ENREF_53)] | 1 | 20yo male with AOSD | Anakinra 100 mg/day + CS | Hepatoxicity (fever, jaundice, coagulation perturbation, and liver enzyme elevations) noted 3 months after starting anakinra  Anakinra stopped and CS dose increased  Gradual recovery to normal liver function 5 months after anakinra discontinued |
| Banse et al. 2013 [[54](#_ENREF_54)] | 1 | 49yo female with AOSD refractory to CS, MTX and infliximab; relapsed during treatment with anakinra; developed melanoma during treatment with tocilizumab | Canakinumab | MAS developed 10 days after second injection  Recovery after treatment with CS + IVIg |
| Eriksson et al. 2013 [[28](#_ENREF_28)] | 1 | 32yo male with AOSD phenotype but CAPS genotype | Anakinra 100 mg/day + CS | Severe local skin reactions accompanied by elevated CRP and ferritin levels  Anakinra discontinued  Patient eventually was successfully treated with canakinumab after poor responses to (1) etanercept and (2) tocilizumab |
| Ahmed et al. 2015 [[55](#_ENREF_55)] | 1 | 46yo female with AOSD | Anakinra 100 mg/day + CS | Liver enzyme levels were elevated 2 weeks after starting anakinra  Anakinra discontinued but restarted when arthralgias recurred  Hospitalised 1 week later with acute hepatotoxicity  Anakinra replaced with etanercept and she recovered |
| Arens et al. 2015 [[56](#_ENREF_56)] | 1 | 29yo female with AOSD | Anakinra 200 mg/day | Pain, erythema, pruritus, and palpable masses at injection sites (appearing as granuloma on FDG-PET) during treatment with anakinra 100 or 200 mg/day  Treatment and outcome not reported |
| Michailidou et al. 2015 [[39](#_ENREF_39)] | 1 | 31yo male with AOSD | Anakinra 100 mg/day + CS | Patient developed new rash that may have been due to AOSD or to anakinra |
| Taylor et al. 2016 [[57](#_ENREF_57)] | 1 | 16yo with AOSD | Anakinra 200 mg/day + CS + naproxen | Drug-induced liver injury marked by elevated liver enzymes + symptoms of encephalopathy; anakinra dose reduced to 100 mg/day then discontinued  Patient made full recovery within 1 month of anakinra withdrawal |
| Bilgin et al. 2018 [[58](#_ENREF_58)] | 1 | 46yo male with AOSD refractory to multiple drugs, including CS, conventional DMARDs (hydroxychloroquine, MTX, leflunomide sulfasalazine), anti-TNF agents (adalimumab, etanercept, infliximab) and tocilizumab | Anakinra | Remission for 1 year while receiving anakinra, at which time the patient developed *Aspergillus* pneumonia (successfully treated with amphotericin B) |

AOSD, adult-onset Still’s disease; ARDS, acute respiratory distress syndrome; CS, corticosteroids; DMARDs, disease-modifying antirheumatic drugs; FDG-PET, Fluorodeoxyglucose positron emission tomography; ICU, intensive care unit; IVIg, intravenous immunoglobulins; MAS, macrophage activation syndrome; MTX, methotrexate; NUD, neutrophilic urticarial dermatosis; NSAID, nonsteroidal anti-inflammatory drug; PCU, progressive care unit; SIRS, severe systemic inflammatory response syndrome; TNF, tumor necrosis factor; yo, years old.

# References

1. Rudinskaya A, Trock DH. Successful treatment of a patient with refractory adult-onset still disease with anakinra. J Clin Rheumatol. 2003;9:330–2.

2. Vasques Godinho FM, Parreira Santos MJ, Canas da Silva J. Refractory adult onset Still's disease successfully treated with anakinra. Ann Rheum Dis. 2005;64:647–8.

3. Goldbach-Mansky R, Wilson M, Snyder C. Differential response to the long acting IL-1 inhibitor IL-1 TRAP in 2 patients with adult onset Still's disease. Ann Rheum Dis. 2006;65:262.

4. Kotter I, Wacker A, Koch S, Henes J, Richter C, Engel A, et al. Anakinra in patients with treatment-resistant adult-onset Still's disease: four case reports with serial cytokine measurements and a review of the literature. Semin Arthritis Rheum. 2007;37:189–97.

5. Mubashir E, Ahmed MM, Hayat S, Heldmann M, Berney SM. Pulmonary hypertension in a patient with adult-onset Stills disease. Clin Rheumatol. 2007;26:1359–61.

6. Debiais S, Maillot F, Luca L, Buret J, Fautrel B, Renard JP. Efficacy of anakinra in a case of refractory Still disease. J Clin Rheumatol. 2008;14:357–8.

7. Gesierich A, Stoevesandt J, Kneitz C, Brocker EB, Schon MP. Adult-onset Still's disease: an uncommon differential diagnosis of urticaria and treatment with anakinra. J Eur Acad Dermatol Venereol. 2009;23:104–6.

8. Mylona E, Golfinopoulou S, Samarkos M, Fanourgiakis P, Papadakos V, Skoutelis A. Acute hepatitis in adult Still's disease during corticosteroid treatment successfully treated with anakinra. Clin Rheumatol. 2008;27:659–61.

9. Priori R, Ceccarelli F, Barone F, Iagnocco A, Valesini G. Clinical, biological and sonographic response to IL-1 blockade in adult-onset Still's disease. Clin Exp Rheumatol. 2008;26:933–7.

10. De Bandt M, Saint-Marcoux B. Tocilizumab for multirefractory adult-onset Still's disease. Ann Rheum Dis. 2009;68:153–4.

11. El Karoui K, Karras A, Lebrun G, Charles P, Arlet JB, Jacquot C, et al. Thrombotic microangiopathy and purtscher-like retinopathy associated with adult-onset Still's disease: a role for glomerular vascular endothelial growth factor? Arthritis Rheum. 2009;61:1609–13.

12. Sentic M, Baresic M, Anic B, Bosnic D, Mayer M, Cerovec M, et al. Treatment challenges in patient with refractory adult Still's disease - a case report. In: XIII Mediterranean Congress of Rheumatology. Cavtat, Croatia; 2009.

13. Lahiri M, Teng GG. A case of refractory adult-onset Still's disease treated with anakinra. Int J Rheum Dis. 2010;13:e36–41.

14. Moulis G, Sailler L, Astudillo L, Pugnet G, Arlet P. May anakinra be used earlier in adult onset Still disease? Clin Rheumatol. 2010;29:1199–200.

15. Perdan-Pirkmajer K, Praprotnik S, Tomsic M. A case of refractory adult-onset Still's disease successfully controlled with tocilizumab and a review of the literature. Clin Rheumatol. 2010;29:1465–7.

16. Quartuccio L, Maset M, De Vita S. Efficacy of abatacept in a refractory case of adult-onset Still's disease. Clin Exp Rheumatol. 2010;28:265–7.

17. Tamaki H, Shimizu H, Hiraoka E, Uechi E, Kishimoto M, Yamaguchi K, et al. Marked effect and steroid-sparing ability of anakinra on a patient with refractory adult-onset Still's disease. Mod Rheumatol. 2010;20:200–4.

18. Agarwal S, Moodley J, Ajani Goel G, Theil KS, Mahmood SS, Lang RS. A rare trigger for macrophage activation syndrome. Rheumatol Int. 2011;31:405–7.

19. Brusch A, Hollingsworth P, Mclean-Tooke A. Successful treatment of refractory adult onset Still's disease with anakinra. In: Annual Meeting of the Australasian Society of Clinical Immunology and Allergy (ASCIA). Sydney, Australia; 2011.

20. Fischer-Betz R, Specker C, Schneider M. Successful outcome of two pregnancies in patients with adult-onset Still's disease treated with IL-1 receptor antagonist (anakinra). Clin Exp Rheumatol. 2011;29:1021–3.

21. Raffeiner B, Botsios C, Dinarello C, Ometto F, Punzi L, Ramonda R. Adult-onset Still's disease with myocarditis successfully treated with the interleukin-1 receptor antagonist anakinra. Joint Bone Spine. 2011;78:100–1.

22. Rech J, Ronneberger M, Englbrecht M, Finzel S, Katzenbeisser J, Manger K, et al. Successful treatment of adult-onset Still's disease refractory to TNF and IL-1 blockade by IL-6 receptor blockade. Ann Rheum Dis. 2011;70:390–2.

23. Jaqua NT, Finger D, Hawley JS. Adult-onset Still's disease masquerading as sepsis in an asplenic active duty soldier. Case Rep Med. 2012;2012:349521.

24. Kontzias A, Efthimiou P. The use of Canakinumab, a novel IL-1beta long-acting inhibitor, in refractory adult-onset Still's disease. Semin Arthritis Rheum. 2012;42:201–5.

25. Loh NK, Lucas M, Fernandez S, Prentice D. Successful treatment of macrophage activation syndrome complicating adult Still disease with anakinra. Intern Med J. 2012;42:1358–62.

26. Petryna O, Cush JJ, Efthimiou E. IL-1 Trap rilonacept in refractory adult onset Still's disease. Ann Rheum Dis. 2012;71:2056–7.

27. Rech J, Manger B, Lang B, Schett G, Wilhelm M, Birkmann J. Adult-onset Still's disease and chronic recurrent multifocal osteomyelitis: a hitherto undescribed manifestation of autoinflammation. Rheumatol Int. 2012;32:1827–9.

28. Eriksson P, Jacobs C, Soderkvist P. A patient with a phenotype of adult-onset still disease, but a genotype typical of cryopyrin-associated periodic fever syndrome. J Rheumatol. 2013;40:1632–3.

29. Fischbach KE, Coe B, Radwan M, Rodriguez J, Carter J, Rumbak MJ. Macrophage activation syndrome in a patient with adult Still's disease following rituximab: presenting with "pseudo-septic shock". ICU Director. 2013;4:248–51.

30. Orr J, Bury Y, Hudson M, Masson S. Liver transplantation for acute liver failure caused by macrophage activation syndrome. Transpl Int. 2013;26:e105–8.

31. Athanassiou P, Basdragianni D, Tzanavari A, Triantafyllidou E, Kostoglou-Athanassiou I. Adult Still's disease: successful treatment with canakinumab. Osteoporos Int. 2014;25:S424.

32. Barsotti S, Neri R, Iacopetti V, d'Ascanio A, Talarico R, Tripoli A, et al. Successful treatment of refractory adult-onset Still disease with canakinumab: a case report. J Clin Rheumatol. 2014;20:121.

33. Choi AD, Moles V, Fuisz A, Weissman G. Cardiac magnetic resonance in myocarditis from adult onset Still's disease successfully treated with anakinra. Int J Cardiol. 2014;172:e225–7.

34. Gutmark R, Eliasieh K, Rivera-Michlig R. A case of bilateral trochleitis in adult-onset Still's disease. Semin Arthritis Rheum. 2014;43:689–91.

35. Hartig I, Schroeder JO, Mrowietz U. Neutrophilic urticarial dermatosis (NUD) in probable adult-onset Still disease responding to anakinra. J Clin Rheumatol. 2014;20:96–8.

36. Lo Gullo A, Caruso A, Pipitone N, Macchioni P, Pazzola G, Salvarani C. Canakinumab in a case of adult onset still's disease: efficacy only on systemic manifestations. Joint Bone Spine. 2014;81:376–7.

37. Yilmaz S, Cinar M, Simsek I, Erdem H, Pay S. Long-term efficacy and safety of Anakinra in a patient with liver transplant due to Adult onset Still's Disease. Mod Rheumatol. 2014;24:1030–1.

38. Luconi N, Risse J, Busato T, Galland J, Mandry D, Voilliot D, et al. Myocarditis in a young man with adult onset Still's disease successfully treated with Il-1 blocker. Int J Cardiol. 2015;189:220–2.

39. Michailidou D, Shin J, Forde I, Gopalratnam K, Cohen P, DeGirolamo A. Typical evanescent and atypical persistent polymorphic cutaneous rash in an adult Brazilian with Still's disease: a case report and review of the literature. Auto Immun Highlights. 2015;6:39–46.

40. Waghmare S, Valecka B, Cairns AP. A severe case of adult onset Stills disease with myopericarditis, resistant to treatment with tocilizumab but responsive to anakinra. Ulster Med J. 2015;84:130–2.

41. Agnihotri A, Ruff A, Gotterer L, Walker A, McKenney AH, Brateanu A. Adult onset Still's disease associated with Mycoplasma pneumoniae infection and hemophagocytic lymphohistiocytosis. Case Rep Med. 2016;2016:2071815.

42. Kumar A, Kato H. Macrophage activation syndrome associated with adult-onset Still's disease successfully treated with anakinra. Case Rep Rheumatol. 2016;2016:3717392.

43. Mehta MV, Manson DK, Horn EM, Haythe J. An atypical presentation of adult-onset Still's disease complicated by pulmonary hypertension and macrophage activation syndrome treated with immunosuppression: a case-based review of the literature. Pulm Circ. 2016;6:136–42.

44. Minori JJ, Wieseltier EH, Benjamin C, Lawler MH. Regaining independence in adult onset Still's disease: a case report. In: Annual Assembly of the American Academy of Physical Medicine and Rehabilitation. New Orleans, LA; 2016.

45. Nataraja C, Griffiths H. Atypical cutaneous manifestations in adult onset still's disease. Case Rep Rheumatol. 2016;2016:4835147.

46. Yuhua W, Zhao M. The first case report of successful treatment of refractory adult-onset still disease with anakinra in China. In: 18th Asia Pacific League of Associations for Rheumatology Congress (APLAR 2016). Shanghai, China; 2016.

47. Parisi F, Paglionico A, Varriano V, Ferraccioli G, Gremese E. Refractory adult-onset Still disease complicated by macrophage activation syndrome and acute myocarditis: a case report treated with high doses (8 mg/kg/d) of anakinra. Medicine (Baltimore). 2017;96:e6656.

48. Albersmeyer MP, Hilge RG, Schulze-Koops H, Sitter T. Adult-onset Still's disease in a patient with cystic fibrosis and its successful treatment with anakinra. Rheumatology (Oxford). 2012;51:1730–2.

49. Breillat P, Tourte M, Romero P, Hayem G, Padovano I, Costantino F, et al. Interleukin-1 Inhibitors and dacryoadenitis in adult-onset Still disease. Ann Intern Med. 2018;168:455–6.

50. Farooq Sheikh AS, Marks J, Hopkinson N. Adult-onset Still's disease with prominent polyserositis. Scott Med J. 2018;63:63–6.

51. Piel-Julian ML, Moulis G, Fournier P, Dupont R, Geiger D, Astudillo L, et al. Early use of anakinra in adult-onset Still's disease myocarditis. Scand J Rheumatol. 2018;47:511–2.

52. Guignard S, Dien G, Dougados M. Severe systemic inflammatory response syndrome in a patient with adult onset Still's disease treated with the anti-IL1 drug anakinra: a case report. Clin Exp Rheumatol. 2007;25:758–9.

53. Aly L, Iking-Konert C, Quaas A, Benten D. Subacute liver failure following anakinra treatment for adult-onset Still disease. J Rheumatol. 2013;40:1775–7.

54. Banse C, Vittecoq O, Benhamou Y, Gauthier-Prieur M, Lequerre T, Levesque H. Reactive macrophage activation syndrome possibly triggered by canakinumab in a patient with adult-onset Still's disease. Joint Bone Spine. 2013;80:653–5.

55. Ahmed O, Brahmania M, Alsahafi M, Alkhowaiter S, Erb S. Anakinra hepatotoxicity in a patient with adult-onset Still's disease. ACG Case Rep J. 2015;2:173–4.

56. Arens AI, Vriens D, Janssen M, Simon A, Oyen WJ. Anakinra injection site reaction on FDG PET/CT. Clin Nucl Med. 2015;40:492–3.

57. Taylor SA, Vittorio JM, Martinez M, Fester KA, Lagana SM, Lobritto SJ, et al. Anakinra-induced acute liver failure in an adolescent patient with Still's disease. Pharmacotherapy. 2016;36:e1–4.

58. Bilgin E, Erden A, Kilic L, Sari A, Armagan B, Kalyoncu U, et al. Aspergillus pneumonia in a patient with adult-onset Still disease successfully treated with anakinra. J Clin Rheumatol. 2018;24:156–8.
